# Deciphering microbial and metabolic influences in gastrointestinal diseases-unveiling their roles in gastric cancer, colorectal cancer, and inflammatory bowel disease

**DOI:** 10.1186/s12967-025-06552-w

**Published:** 2025-05-16

**Authors:** Daryll Philip, Rebecca Hodgkiss, Swarnima Kollampallath Radhakrishnan, Akshat Sinha, Animesh Acharjee

**Affiliations:** 1 Cancer and Genomic Sciences, School of Medical Sciences, College of Medicine and Health, University of Birmingham Dubai, Dubai, UAE; 2 Cancer and Genomic Sciences, School of Medical Sciences, College of Medicine and Health, University of Birmingham, Birmingham, UK; 3 Centre for Health Data Research, University of Birmingham, Birmingham, UK; 4 Institute of Translational Medicine, University Hospitals Birmingham NHS, Foundation Trust, Birmingham, UK

**Keywords:** Gastric cancer, Inflammatory bowel disease, Colorectal cancer, Microbiome, Metabolome, Biomarkers, Machine learning

## Abstract

**Introduction:**

Gastrointestinal disorders (GIDs) affect nearly 40% of the global population, with gut microbiome-metabolome interactions playing a crucial role in gastric cancer (GC), colorectal cancer (CRC), and inflammatory bowel disease (IBD). This study aims to investigate how microbial and metabolic alterations contribute to disease development and assess whether biomarkers identified in one disease could potentially be used to predict another, highlighting cross-disease applicability.

**Methods:**

Microbiome and metabolome datasets from Erawijantari et al. (GC: n = 42, Healthy: n = 54), Franzosa et al. (IBD: n = 164, Healthy: n = 56), and Yachida et al. (CRC: n = 150, Healthy: n = 127) were subjected to three machine learning algorithms, eXtreme gradient boosting (XGBoost), Random Forest, and Least Absolute Shrinkage and Selection Operator (LASSO). Feature selection identified microbial and metabolite biomarkers unique to each disease and shared across conditions. A microbial community (MICOM) model simulated gut microbial growth and metabolite fluxes, revealing metabolic differences between healthy and diseased states. Finally, network analysis uncovered metabolite clusters associated with disease traits.

**Results:**

Combined machine learning models demonstrated strong predictive performance, with Random Forest achieving the highest Area Under the Curve(AUC) scores for GC(0.94[0.83–1.00]), CRC (0.75[0.62–0.86]), and IBD (0.93[0.86–0.98]). These models were then employed for cross-disease analysis, revealing that models trained on GC data successfully predicted IBD biomarkers, while CRC models predicted GC biomarkers with optimal performance scores.

**Conclusion:**

These findings emphasize the potential of microbial and metabolic profiling in cross-disease characterization particularly for GIDs, advancing biomarker discovery for improved diagnostics and targeted therapies.

**Supplementary Information:**

The online version contains supplementary material available at 10.1186/s12967-025-06552-w.

## Introduction

Gastrointestinal diseases (GIDs) are disorders that impact the gastrointestinal tract, which extends from the esophagus to the rectum and includes the pancreas, liver, and gallbladder [1]. GIDs can be broadly categorized into those resulting from malignancies, such as gastric cancer (GC), pancreatic cancer, esophageal cancer, liver cancer, and colorectal cancer, also known as colon cancer (CRC) [2], and those driven by inflammatory responses, including inflammatory bowel disease (IBD) and irritable bowel syndrome (IBS) [3].

Among malignancy related GIDs, GC represents a significant global health burden. The primary risk factor for GC is infection by *Helicobacter pylori* (*H. pylori*), which causes chronic inflammation and significantly elevates the risk of malignant tumor formation in the gastric lining [4]. According to the Global Cancer Observatory (GLOBOCAN) 2022 data, GC is the fifth most common cancer worldwide, with 968,350 new cases and 659,853 deaths reported [5]. It ranks among the leading causes of mortality in 42 countries, with higher incidence rates in males compared to females, particularly in regions of Eastern Asia [6].

Likewise, CRC is characterized by malignant growths or polyps in the colon or rectum [7]. According to GLOBOCAN, CRC ranks as the third most commonly diagnosed cancer and the second leading cause of cancer related deaths globally, with 1.9 million new cases and 903,859 deaths in 2022. The disease is more prevalent in men than women, with the highest incidence rates found in European countries such as Norway and Denmark [5].

In contrast, inflammation driven GIDs, such as IBD, are characterized by chronic morbidity resulting from immune mediated inflammatory processes. IBD serves as an umbrella term that includes Crohn's disease (CD) and ulcerative colitis (UC) [8, 9]. According to the Global Burden of Disease (GBD) 2019, there were approximately 4.9 million IBD cases worldwide, resulting in 41,000 deaths. Prevalence rates were highest in Norway, followed by Canada, with both prevalence and mortality rates being higher in females compared to males [10].

Established risk factors such as dietary habits [11–13], genetic predispositions [14–16], and lifestyle choices [17–19] are more or less associated with GC, CRC, and IBD. Another significant risk factor is dysbiosis, which refers to an imbalance in the gut microbiome, the vast and diverse community of bacteria and other microorganisms residing in our digestive system. Research shows that the abundance or depletion of certain microbes in the gut, which is often referred to as the ‘second brain’, can play a pivotal role in GIDs [20]. For instance, Zeng et al. [21] demonstrated that in addition to *H.pylori,* microbes like *Prevotella* and *Streptococcus* were abundant, while beneficial microbes such as *Bifidobacterium* were depleted in the fecal samples from GC patients.

Similarly, Villéger et al. [22] reported increased levels of *Bacteroides* and *Prevotella* in CRC, alongside reduced levels of *Lactobacillus* and *Faecalibacterium*. In IBD, *Streptococcus* levels were elevated in UC patients, and *Lachnoclostridium* and *Fusobacterium* were significantly increased in CD patients [23].

Beyond microbial composition, GIDs are influenced by the metabolites produced by the gut microbiome. Disruptions in microbial metabolite production can lead to metabolic reprogramming, contributing to GC, IBD, and CRC pathogenesis. For example, metabolic pathways involving lipids, nucleotides, and amino acids such as alanine and valine were found to be dysregulated in patients with GC [24, 25]. Zhang et al. [26] highlighted that microbiota derived metabolites such as trimethylamine-N-oxide, secondary bile acids, hydrogen sulfide, and N-nitroso compounds could induce inflammation and modulate tumor immunity in the colon. In IBD, bile acids, such as deoxycholic acid, activate inflammatory signaling pathways while dysregulated tryptophan metabolism, particularly in UC patients, further heightens intestinal inflammation [27, 28].

GIDs are serious conditions with high mortality and morbidity rates, often going undiagnosed in their early stages [29]. This is partly because the symptoms can be subtle or easily overlooked. Unfortunately, delayed diagnosis allows these diseases to progress rapidly, with one condition often leading to another through shared pathological mechanisms.

For instance, Fretwell et al. [30], in their systemic review of case reports containing histopathology data, stated that although rare, GC can metastasize to the colorectum through lymphatic, vascular, or mesenteric routes.

Similarly, Tak et al. [31] analyzed clinicopathological characteristics of patients in Korea and reported that individuals with CRC are at an increased risk of developing intestinal metaplasia and gastric adenomas, which are precursors to GC, within the first four years of CRC diagnosis. Furthermore, Sato et al. [32] emphasized in their study, which evaluated clinical studies, meta-analyses, and systematic reviews, that chronic mucosal inflammation in UC can increase the chances of developing colorectal neoplasia by progressing from low-grade to high-grade dysplasia, eventually developing into CRC.

Given the complexity of microbiome-metabolite interactions and their critical role in GIDs, it has become steadily more beneficial to train machine learning models to produce highly accurate, reproducible, and interpretable insights from large and complex datasets [33]. Recent studies highlight the effectiveness of machine learning algorithms in differentiating diseased patients from healthy individuals [34, 35], detecting important microbial and metabolic biomarkers [36–39], and uncovering risk factors [40] associated with diseases that affect the gastrointestinal tract.

Our goal is to implement machine learning models for GC, IBD, and CRC to identify the most significant and differential microorganisms and metabolites in fecal samples using publicly available datasets. These biomarkers are then used to stratify and predict cross-disease associations. Specifically, the GC model was utilised to predict IBD and CRC, the IBD model was used to predict GC and CRC, and the CRC model was applied to predict GC and IBD. This approach allows us to uncover both shared and unique patterns among GIDs. Subsequently, the identified biomarkers are integrated into in-silico modeling techniques to assess microbial contributions to metabolite production. Finally, network analysis techniques are conducted to uncover correlations between features and biological pathways linking microbes and metabolites in diseased and healthy patients.

## Methods

### Data preprocessing

To mitigate overfitting and improve model performance, we eliminated sparse features from the dataset, and the remaining data was normalized using min–max scaling [41], transforming values to a range between 0 and 1. This prevented the dominance of features with larger ranges or values, ensuring that all the features contribute equally to the model.

### Unsupervised machine learning models

#### Principal components analysis (PCA)

We applied PCA [42] for dimensionality reduction, identifying principal components (PC1 and PC2) that capture maximum variance for the metabolite datasets. Outliers were detected by calculating the Mahalanobis distance [43], with a 95% confidence interval threshold derived from the chi-squared distribution.

#### Principal coordinates analysis (PCoA)

We performed PCoA [44], or metric multidimensional scaling, for outlier detection on the microbiome datasets. The Bray–Curtis [45] dissimilarity matrix was calculated based on abundance data, and the resulting PCoA plot projected the high-dimensional data into a lower-dimensional space. This visualisation captured similarities and differences between samples using the first two principal coordinates. The 95% confidence ellipses for each group provide a visual means of identifying potential outliers by highlighting variations within and between sample groups.

### Univariate statistical analysis

To prioritize the most promising features for the computationally intensive machine learning models, we applied non-parametric tests such as the Mann–Whitney U test [46] for GC and IBD and the Kruskal–Wallis H test [47] for CRC to assess differences between two independent groups, particularly when the data does not follow normal distribution. To control the rate of false positives, p-values were adjusted using the Benjamini-Hochberg (BH) [48] method. To optimize computational efficiency, we considered features with adjusted p-values below 0.05 as significant, focusing our analysis on only the most differential microbes and metabolites.

### Supervised machine learning models

Three machine learning models were used in this work to analyze the microbiome and metabolome datasets efficiently. The workflow can be seen in Supplementary Fig. 1. We employed eXtreme gradient boosting (XGBoost) [49] as an ensemble algorithm that excels at classification tasks by iteratively refining predictions through gradient boosting. It incorporates regularization techniques to minimize errors and prevent overfitting, ensuring a balance between accuracy and model complexity. Similarly, Random Forest [50] was employed due to its ability to handle high-dimensional microbiome data. It reduces overfitting and boosts overall performance by constructing numerous decision trees using random subsets of the input, thus reducing variance and improving model stability. In addition to the ensemble models, the Least Absolute Shrinkage & Selection Operator (LASSO) [51] was utilised due to its ability to combine classification and feature selection. In binary classification, LASSO uses the regularization parameter C to control regularization strength. Since C is the inverse of λ, which is the penalty term coefficient (C = 1/λ), a higher C value reduces regularization, allowing more features to stay in the model. Conversely, a lower C value strengthens regularization, shrinking more coefficients to zero. This eliminates the need for a separate feature selection method for LASSO.

### Hyperparameter tuning through random search and Bayesian optimization

Hyperparameters are settings that control the behavior of machine learning algorithms, and hyperparameter tuning optimizes model performance by finding the best settings. In our study, this process begins with a random search [52], which explores random combinations of hyperparameters. However, this approach may miss optimal feature combinations if the search space is sparse.

To address this, Bayesian optimization (BO) [53] was employed. BO used a Gaussian process to build a probabilistic model from the best random search results, guiding the selection of subsequent hyperparameters. In this study, the data was split 75% for training and 25% for testing.

The random search was followed by fivefold cross-validation to refine the parameter grid, which was then optimized using BO with fivefold cross-validation to identify the best-performing hyperparameters, which were initially applied on the training data and then on the unseen test data.

### Model evaluation

Performance metrics, like Receiver Operating Characteristic Area Under Curve (ROC-AUC) [54], were calculated across the models for all the diseases in our study because of their ability to consider trade-offs between specificity and recall, with higher values indicating better discrimination. Alongside ROC-AUC, other metrics such as accuracy, precision, recall, F1-score, and specificity were calculated, which provided a more comprehensive evaluation of the models. To further assess the reliability of these scores, the 95% confidence intervals (CI) were computed for each performance metric.

### Feature selection

We applied Recursive Feature Elimination with Cross-Validation (RFECV) to XGBoost and Random Forest to refine the feature sets. This process iteratively removed less important features through cross-validation, selecting the optimal set based on the highest cross-validation scores. Overlapping features selected by RFECV and those automatically chosen by LASSO were identified. However, since the number of microbes and metabolites remained high, we further evaluated feature subsets by computing ROC-AUC scores for the best performing models. Subsets with the top 5, 10, 15, 20, 25, and 30 features were iteratively tested, and the model achieving the highest AUC with the fewest features was chosen for all diseases. Feature rankings were determined using either LASSO coefficients or Gini feature importance. Finally, a Spearman correlation cluster map with hierarchical clustering was generated to visualise clusters of microbes and metabolites that were strongly correlated, which provided more understanding into their relationships.

### Diversity analysis

To understand the complexity and diversity of the microbial communities in the gut within the healthy and diseased groups, we measured alpha diversity [55] indices, which quantify both richness (the number of distinct genera) and evenness (the uniformity of distribution among those genera). The diversity index (D) value increases with higher richness and evenness. Among these, the Shannon-Weiner index [56] is the most used metric due to its ease of interpretation. It measures the uncertainty of predicting a species from a community and is particularly sensitive to species richness.

The probability that two randomly chosen microbes belong to the same species is measured by Simpson's Index [57] with lower values indicating greater diversity. It is often expressed as the Gini-Simpson index (1 − D). Statistical significance within groups was evaluated by calculating p-values and applying FDR correction.

Beta diversity [58] captures the variation or dissimilarity in genera between the sample groups. We utilised the non-metric dimensional scaling (NMDS) to visualise the similarities or dissimilarities in a low-dimensional space, employing the Jaccard distance. The stress values obtained from NMDS indicate the accuracy of 2D representations with lower stress values indicating a better fit between the original dissimilarity matrix and the NMDS ordination (see Table 1).

**Table 1:** Summary of datasets used for training and validation in GC, CRC, and IBD

Datasets	Source	Healthy	Diseased	Total features	Source of extraction	Technology used
Training data						
GC						
Microbiome	Erawijantari et al. [178]	54	42	10,528	Fecal	Shotgun metagenomics sequencing
Metabolome				525		*CE-TOFMS
CRC						
Microbiome	Yachida et al. [179]	127	150	11,942	Fecal	Whole genome sequencing
Metabolome				450		CE-TOFMS
IBD						
Microbiome	Franzosa et al. [180]	56	164	11,720	Fecal	Whole genome shotgun sequencing
Metabolome				466		*LC–MS
Validation data						
GC						
Microbiome	Jaeyun Sung et al. [181]	10	40	470	Gastric antrum	16S rRNA sequencing
Metabolome	UK BioBank	44,378	2,436	168	Plasma	NMR spectroscopy
CRC						
Microbiome	Kim et al. [182]	102	36	499	Fecal	16S rRNA sequencing
Metabolome				462		*UPLC-MS/MS
IBD						
Microbiome	iHMP/HMP2 [183]	104	278	9694	Fecal	Shotgun metagenomic sequencing
Metabolome				596		LC–MS

This table includes the total number of healthy and diseased patients, the number of microbiome and metabolome features, the source of extraction for microbes and metabolites, and the sequencing and analytical tools employed, respectively

*CE-TOFMS: Capillary electrophoresis time-of-flight mass spectrometry, *LC–MS: liquid chromatography-mass spectrometry, *UPLC-MS/MS: ultra-performance liquid chromatography-mass spectrometry

### Microbial community model (MICOM)

To explore connections between the microbes and metabolites identified, a microbial community model was created for each disease, stimulating gut communities for each sample. MICOM uses an L2 normalisation based model to calculate the community growth rate, denoted as ${\mu }_{c}$, for all the microbes in a metagenomic sample [59]. This method enables what Diener et al. define as selfish individual growth maximization [59], allowing each microbe to reach its maximal growth (${\mu }_{i})$ rather than just a maximal overall community growth. Simulated growth rates are determined based on the microbes relative abundance, known metabolite fluxes, and growth rates from an input database, as well as user input minimal and maximal abundance values and growth rates. Utilizing the relative abundance of each genus selected by the machine learning process and its corresponding classification, a manifest for each disease was built using the build function from micom.workflows. The model database was set to the “agora103_genus.qza” [60] dataset, with the solver set to “osqp”, a cutoff equal to zero, threads equal to two, and a phenotype column indicating the disease status for each sample. This manifest is a data frame created by the model that includes all the information on the microbes identified in the provided database, which is then used to construct the growth model.

To obtain maximal growth rates, a cooperative trade-off value must be determined. The model fixes the community growth rate to a fraction of its optimum and then calculates the minimum L2 normalisation of the individual growth rates. Individual growth rates are calculated as follows:

$${\mu }_{i}=\frac{\alpha {\mu }_{c}}{{a}^{T}a}{a}_{i}$$

where α denotes the specified trade-off value (the fraction of community maximum to use), ${\mu }_{c}$ denotes the community growth rate, ${\mu }_{i}$ the individual growth rate for genus i and ${a}_{i}$ is the relative abundance of that genus. Thus, the community growth rate is represented by the sum of individual growth rates and their abundance:

$${\mu }_{C}= {\sum }_{i}{a}_{i}{\mu }_{i}$$

Therefore, before creating the growth model, the optimal value for the trade-off was identified using the resulting manifest of each disease in the tradeoff function from micom.workflows, with the medium set to the “western_diet_gut.qza” [60] database and threads equal to two. The optimal value was defined as the highest trade-off value where the maximal number of taxa were enabled to grow.

Additionally, MICOM represents the flux balances of the microbes and provides the estimated production and consumption of metabolites by these recognised communities. A linear model based on the COBRApy Python package is utilised, with an assumption of a steady state of all fluxes within the microbes system required. The fluxes ${v}_{i}$ are provided in millimoles per gram per hour (mmol/[gDWh]) and follow the rules:

$$maxmizie {v}_{bm}$$

$$such that \left(s.t\right){S}_{v}=0$$

$$and l{b}_{i}\le {v}_{i}\le u{b}_{i}$$

such that ${v}_{bm}$ is the biomass reaction, which is normalised to produce 1 g of biomass in a unit 1/h, to correspond to the growth rate of the organism. Lower and upper bounds $l{b}_{i}$ and $u{b}_{i}$ are used to impose thermodynamic constraints. To allow a community of fluxes, the following must be considered:

$$maximize {\mu }_{c}= \sum_{i}{a}_{i}{\mu }_{i}$$

$$s.t. "i : Sv=0$$

$${\mu }_{i}={{v}_{i}}^{bm}\ge {{\mu }_{i}}^{min}$$

$$l{b}_{i}\le {v}_{i}\le u{b}_{i}$$

$${l{b}_{i}}^{ex}\le {{{a}_{i}v}_{i}}^{ex}\le u{{b}_{i}}^{ex}$$

$${l{b}_{i}}^{m}\le {{v}_{i}}^{m}\le u{{b}_{i}}^{m}$$

where ${{v}_{i}}^{bm}$ is the biomass flux, ${{\mu }_{i}}^{min}$ is the user-specified minimum growth rate (0 is used in this study), ${{v}_{i}}^{ex}$ is the exchange fluxes with the specified external environment, and lb and ub are the lower and upper bounds. ${{v}_{i}}^{m}$ are the exchanges between the entire community and the gut lumen, so a set metabolite environment representing the gut lumen must also be provided to the model. The “western_diet_gut.qza” database is used in this study. Overall production fluxes are calculated via:

$${{v}_{tot}}^{m }= {\sum }_{i,{{v}_{i}}^{m}>0}{a}_{i}{{v}_{i}}^{m}$$

with ${{v}_{i}}^{m}$ representing an exchange flux for the metabolite m in taxon i and ${{v}_{tot}}^{m}$ the total metabolite fluxes.

Consequently, to obtain the growth model and predict metabolite production and consumption by each genus, the grow function was enforced with the input trade-off set to the determined optimum, the manifest for the disease, and the same “western_diet_gut.qza” medium.

From these estimated fluxes, we utilise the phenotype provided for each sample to examine how these fluxes vary across disease groups, using MICOM’s built in non-parametric tests for each metabolite against the phenotype. To identify metabolites differentially produced between case and control samples of each disease, the plot_association function from micom.viz was populated with the growth results, variable_type set to binary, phenotype set to the disease status (case vs control), and fdr_threshold set to 0.5. Any metabolites or their derivatives identified by the MICOM model and in the predictive analytics were noted as important. Finally, the selected genus names were entered into the MicrobiomeAnalyst taxon set analysis tool to identify literature validated interactions between microbes and metabolites and were compared to the results provided by MICOM and machine learning analysis.

### Weighted co-gene network analysis (WCGNA)

WCGNA [61] was performed on both the metabolite and microbiome datasets to explore co-expression patterns and their associations with case–control traits. First, the optimal power to create a scale-free topology network was determined by evaluating a range of soft-thresholding powers ranging from 1 to 20, with plots of mean connectedness and scale independence guiding the decision. Using the chosen power, hierarchical clustering and dynamic tree-cut techniques were used to identify modules of co-expressed features, each assigned a distinct colour for visualisation. For each module, the module eigengenes (MEs), were computed and compared to the case–control trait using the Pearson correlation coefficient. The statistical significance of the correlation coefficients, which varied from −1 (strong negative correlation) to + 1 (strong positive correlation), was assessed using the appropriate p-values obtained from the correlation analysis. A heatmap of these correlations, displaying both the coefficients and the corresponding p-values, provided a comprehensive view of the module-trait relationships.

Additionally, the Topological Overlap Matrix (TOM) was calculated to assess the similarity between features based on their network connectivity. Heatmaps and network dendrograms were created using this TOM to show co-expression patterns. The co-expression networks were shown using graph based visualisation approaches after the TOM was filtered to highlight the strongest linkages for network visualisation.

Features (metabolites or taxa) were represented by nodes in these networks, while co-expression relationships were represented by edges, whose attributes were proportionate to the strength of the connection.

## Results

### Demographic characteristics for all datasets

Given the large number of datasets, we focused on basic demographic characteristics (Table 2). The datasets from Erawijantari et al. (GC) and Yachida et al. (CRC) revealed a higher proportion of male participants compared to females. The median age was 66 years for GC patients and 64 years for CRC, suggesting that the risk of developing GC and CRC may increase with age. However, statistical analysis showed no significant differences in median age between GC patients (p = 0.75), CRC patients (p = 0.14), and healthy controls. BMI for gastrectomy patients was higher (23.2) compared to the healthy group (21), with a p-value of 0.0004, indicating a statistically significant difference between the two groups. However, even though the mean BMI for CRC patients (23) was higher than the healthy group (22.9), there was no statistical difference between the two groups (p = 0.66).

**Table 2:** Baseline demographic characteristics

Population demographics	Gastric cancer (Erawijantari et al.)				Inflammatory bowel disease (Franzosa et al.)				Colerectral cancer (Yachida et al.)			
	Total	Gastrectomy	Healthy	p-value	Total	IBD	Healthy	p-value	Total	CRC	Healthy	p-value
Participants	96	42	54		220	164	56		277	150	127	
Sex												
Male (%)	66.7	33.33	33.33	0.12	NA	NA	NA	NA	57.8	32.1	25.6	0.65
Female (%)	33.3	10.42	22.92		NA	NA	NA		42.2	22	20.2	
Age (median)	65 (60–68)	66 (60–68)	64 (60–68)	0.75	38 (35–43)	41 (36–47)	32 (30–37)	0.005	65 (64–67)	64 (63–66)	67 (65–69)	0.14
BMI (mean)	22.2 (22–23)	23.2 (22–24)	21 (20–22)	0.0004	NA	NA	NA	NA	23 (23–24)	23 (23–24)	22.9 (22–24)	0.66

Table describing the calculations done on the test datasets

P-values for categorical variables, such as sex, were calculated using Fisher’s exact test, while for continuous variables, such as body mass index(BMI) and age, the Mann–Whitney U test was used

NA, not available

For the IBD dataset by Franzosa et al., gender related data was unavailable, but the median age of IBD patients was 41 years. A significant difference in age between healthy individuals and IBD patients suggests a potential association between age and IBD prevalence (p = 0.005). Demographic characteristics for the validation datasets were also calculated, which can be found in (Supplementary Table 2).

### Data preprocessing for gastric cancer

**Fig. 1:**
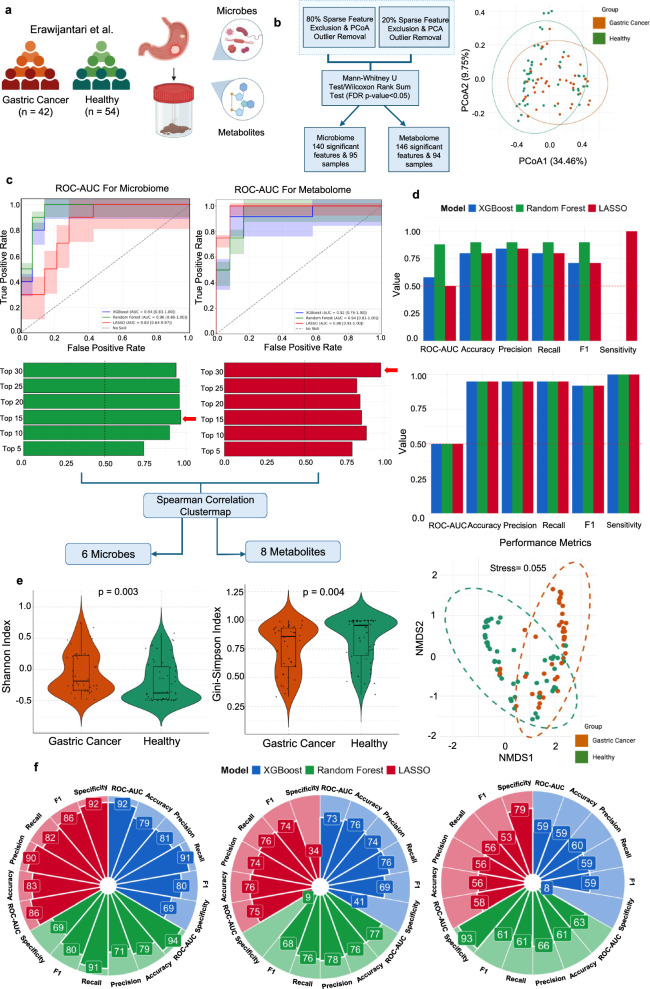
Microbiome-metabolome machine learning for cross-disease predictions in GC. **a** Fecal microbiome and metabolome data from GC patients (orange) and healthy individuals(green) obtained from Erawijantari et al. **b** Data preprocessing workflow highlighting the key microbes, metabolites, and samples selected for machine learning, alongside a principal coordinates analysis (PCoA) plot used for outlier removal. **c** The receiver operator curve – area under the curve (ROC-AUC) for microbiome and metabolome data across models: XGBoost (blue), Random Forest (green), and LASSO (red). Bar graph showing the best-performing model (microbiome-Random Forest, metabolome-LASSO) based on the highest AUC-ROC score, highlighting the optimal number of features. The selection includes 6 microbial and 8 metabolite features identified through Spearman cluster map analysis. **d** Validation performance metrics of the optimal features depicted by bar plots for microbiome and metabolome analysis were evaluated using the microbiome dataset from Jaeyun Sung et al. and the metabolome dataset from the UKBB. **e** Alpha diversity for microbes was visualised with violin plots comparing healthy and GC patients using the Shannon and Gini-Simpson indices. FDR-corrected p-values (p < 0.05) showed significant differences within both groups. Beta diversity was evaluated using non-metric multidimensional scaling (NMDS) based on Jaccard distances, with the stress value confirming statistical significance between healthy and diseased patients. **f** Circular bar plots illustrate the performance scores of the three models trained using combined microbiome and metabolome data from GC patients. Key biomarkers from the GC dataset were identified in the IBD and CRC datasets. GC-trained models were applied to predict IBD and CRC outcomes respectively

In the Erawijantari et al. dataset, we excluded features with 80% sparsity from the microbiome data and those with 20% from the metabolome data. The remaining features were subjected to min–max scaling. During PCoA, we identified one sample in the microbiome data as an extreme outlier based on its visual distance from the main cluster, which was subsequently removed. Similarly, two samples in the metabolome data were identified as outliers based on their visual deviation from the main cluster in the PCA graph. They were excluded, and this resulted in a final dataset of 95 microbiome samples and 94 metabolome samples. Using the Mann–Whitney U test (FDR-adjusted p < 0.05), we identified 140 significant features from the microbiome data and 146 from the metabolome data, which were ultimately used for further analysis (Fig. 1b).

### Model performance across multiple models for gastric cancer and validation

We employed three models, XGBoost, Random Forest, and LASSO, separately on the GC microbiome and metabolome datasets, The models were hyper-tuned through random search and Bayesian optimization. For feature selection, we applied RFECV with tenfold cross-validation for XGBoost and Random Forest, while LASSO utilised its built-in feature selection. This process identified 59 microbes and 45 metabolites that were common across all models, which we used to train the classifiers. Performance metrics were calculated based on test scores.

For the microbiome data, the Random Forest model performed best with an AUC of 0.96 (0.86–1.00) and an accuracy of 88% with the scores and hyper-tuned parameters of the model depicted in Supplementary Tables 3&4.To further reduce the number of microbes, the Gini-importance scores of the microbes were noted, and a subset of the top 15 microbes provided the best ROC-AUC score of 97%.

These microbes at the genus level were subjected to a Spearman correlation cluster map. From the clusters, we identified 6 microbes, mainly *CAG-103*, *Ruminococcus*, *Olleya*, *Cutibacterium*, *Allisonella*, and *Centipeda* (Supplementary Fig. 2a).

For the metabolome data, LASSO was the top performer, with an AUC of 0.98 (0.91–1.00) and an accuracy of 92%. Further feature selection identified a subset of 30 metabolites that had an AUC score of 98%. From the cluster map, 8 metabolites were identified, mainly dihydrouracil, taurine, $\gamma$-butyrobetaine, pimelate, glycocholate, methionine sulfoxide, phenethylamine, and citramalate (Supplementary Fig. 2b) (Fig. 1c).

We choose to validate the top 15 microbes and top 30 metabolites across all three models. We validated the GC microbiome model using data from Jaeyun Sung et al., where Random Forest performed best, with an AUC of 0.88 (0.85–0.99). However, for metabolite validation using the UK Biobank, the AUC scores were lower than expected, with all three models showing an AUC of 0.50 (0.50–0.50) while all other performance metrics remained similar. The reduced AUC scores, despite high sensitivity and recall, are likely due to differences in sample type. The UK Biobank metabolites were derived from plasma samples, whereas the main model was trained using metabolite data extracted from fecal samples. This could have potentially limited the model's predictive power (Supplementary Table 5) (Fig. 1d).

### Microbial diversity & abundance analysis for gastric cancer

Alpha diversity analysis using the selected six microbes revealed significant differences between GC patients post-gastrectomy and healthy individuals. Shannon index (p = 0.003) and Gini-Simpson index (p = 0.004) consistently indicated distinct microbial diversity within the groups. Similarly, beta diversity analysis, using NMDS with Jaccard distances (stress = 0.055), effectively demonstrated microbial differences between the groups (Fig. 1e).

### Using gastric cancer biomarkers to predict inflammatory bowel disease and colorectal cancer

To explore cross-disease applicability, we extended our analysis from GC to include IBD and CRC. We applied the GC model, trained on the selected biomarkers, to predict IBD and classify CRC patients as either diseased or non-diseased (non-IBD and non-CRC). Among the GC models, Random Forest performed the best, achieving a ROC-AUC score of 0.94 (0.83–1.00). Surprisingly, the predictions on both IBD and CRC revealed the Random Forest model as the top-performing model, with an ROC-AUC score of 0.77 (0.71–0.83) and 0.63 (0.57–0.69), respectively (Supplementary Tables 6&7) (Fig. 1f).

### Data preprocessing for colorectal cancer

For preprocessing the CRC dataset, we removed 70% of the sparse features from the microbiome dataset and 80% of the sparse features from the metabolome data as an initial step. After applying min–max scaling to the remaining features, we generated PCoA and PCA plots to identify and remove outliers. This resulted in the exclusion of 10 samples from the microbiome dataset and 12 samples from the metabolome dataset. Since the Wilcoxon test failed to identify significant microbes and metabolites, we applied the Kruskal–Wallis test to filter out insignificant features. Ultimately, 208 microbes across 267 samples and 105 metabolites across 265 samples were used as inputs for the machine learning models (Supplementary Fig. 3).

### Model performance across multiple models for colorectal cancer and validation

We trained hyperparameter-tuned machine learning models on the CRC microbiome and metabolome datasets. Feature selection resulted in 82 microbes and 72 metabolites that were consistently identified across the three models. Among the models built for CRC microbes, Random Forest performed the best, achieving an AUC-ROC score of 0.89 (0.80–0.96) and an accuracy of 84%. However, when we focused on the top 15 microbes, the AUC score dropped significantly to 54%, with other subsets performing even worse. Despite this decrease, we decided to proceed with this subset to strike a balance between model performance and the identification of actionable biomarkers. From the heatmap, we identified 13 microbes such as *HGM04593, Parafilimonas, RUG11977, UMGS755, CACXMZ01, Desulfitobacterium, Onthousia, M0103, Fusobacterium, Enterococcus, Selenomonas, Psychrobacillus*, and *Thermus* (Supplementary Fig. 4a).

For the metabolome data, LASSO performed the best, with an AUC score of 0.70 (0.57–0.82). From the top 15 metabolites, which yielded the highest AUC score of 61%, we selected 10 metabolites such as isoleucine, N6-methyl-2-deoxyadenosine, N1,N8-diacetylspermidine, guanine, $\gamma$-guanidinobutyrate, dTMP, nicotinamide, decanoate, dodecanedioate, and 2-hydroxyoctanoate were selected for final analysis from the heatmap(Supplementary Fig. 4b)(Supplementary Tables 8&9).

To validate the CRC models, we used the microbiome and metabolome from Kim et al. XGBoost and Random Forest achieved an AUC of 0.51 (0.47–0.54) for the microbiome, while LASSO for metabolites achieved an AUC of 0.50 (0.39–0.61). The lower performance could likely stem from differences in particular biomarker profiles or technical variability between datasets (Supplementary Table 10).

### Microbial diversity and abundance analysis For CRC

Alpha diversity indices such as the Shannon index (p = 0.0617) and Gini Simpson (p = 0.524) indicated no significant differences in microbial composition within the healthy and within the diseased groups. Similarly, beta diversity using the NMDS analysis had a stress value of 0.7, further confirming no significant diversity between healthy and diseased samples.

### Using colorectal cancer biomarkers to predict gastric cancer and inflammatory bowel disease

We used the model trained on the selected combined biomarkers for CRC to predict and distinguish GC from non-GC patients and IBD from non-IBD patients. Within the CRC models, Random Forest achieved the highest ROC-AUC score of 0.75 (0.62–0.86). For GC predictions, Random Forest led with a top ROC-AUC of 0.86 (0.77–0.93), whereas for IBD predictions, the LASSO model produced the top AUC score of 0.65 (0.57–0.72) (Supplementary Tables 11, 12).

### Data preprocessing for inflammatory bowel disease

For the IBD data, we removed 40% of sparse features from the microbiome dataset and 60% from the metabolome dataset, followed by min–max scaling of the remaining features. For outlier removal, we used PCoA on the microbiome data, identifying and removing nine outliers, and PCA on the metabolome data, removing seven outliers. After applying the Mann–Whitney U test, we identified 1089 significant microbiome features across 211 samples and 259 metabolome features across 213 samples, which were used for further analysis (Supplementary Fig. 5).

### Model performance across multiple models for inflammatory bowel disease and validation

We individually trained the IBD microbiome and metabolome data using hyperparameter-tuned XGBoost, Random Forest, and LASSO models. Feature selection through RFECV and LASSO consistently identified 83 significant microbiome features and 73 metabolome features across all three models. For the microbiome data, Random Forest demonstrated the best performance, with an AUC of 0.90 (0.81–0.97) and an accuracy of 83%. The subset of the top 15 microbes had a top score of 89%, and the Spearman cluster map identified 9 microbes specifically, *Actinomarina*, *RGIG4708*, *Butyribacter, Limivivens, Faecalibaculum, UBA11774, UMGS1601, Bariatricus*, and *SIG607* (Supplementary Fig. 6a).

For the metabolome data, Random Forest was the top performer, with an AUC of 0.95 (0.89–0.99) and an accuracy of 89%. The subset of 20 metabolites had the top score of 88% and identified 10 metabolites such as urobilin, glycerate, cholestenone, acetyl-arginine, 4-hydroxy 3-methyl acetophenone, methylguanine, pseudouridine, inosine, 1,3,7-trimethyl urate, and carnosol based on the cluster map and Gini-importance scores (Supplementary Fig. 6b, Supplementary Tables 13, 14).

For validation of the IBD models, we used the Integrative Human Microbiome Project (iHMP) datasets, and for the microbes and metabolites, we have Random Forest as the best model with an AUC of 0.60 (0.50–0.64) and AUC of 0.76 (0.70–0.81), respectively. These microbes and metabolites can be investigated as potential biomarkers in IBD alone (Supplementary Table 15).

### Microbial diversity and abundance analysis for inflammatory bowel disease

For IBD, the Shannon diversity index (p = 0.617), and Gini Simpson (p = 0.525) values for alpha diversity indicate that there are no microbial differences within the healthy and IBD groups. However, the beta diversity analysis, visualised through NMDS (stress value = 0.05), indicated distinct compositional differences between the groups.

### Using inflammatory bowel disease biomarkers to predict gastric cancer and colorectal cancer

We used the model trained on the selected combined biomarkers for IBD to predict and distinguish GC from non-GC patients and CRC from non-CRC patients. Among the main IBD models, Random Forest had the highest AUC score of 0.93 (0.86–0.98). For the GC predictions, we noticed that Random Forest had the best AUC score of 0.66 (0.54–0.76), and for predictions on CRC, LASSO had a top score of 0.57 (0.51–0.63) (Supplementary Tables 16, 17).

### Interactions between microbes and metabolites

**Fig. 2:**
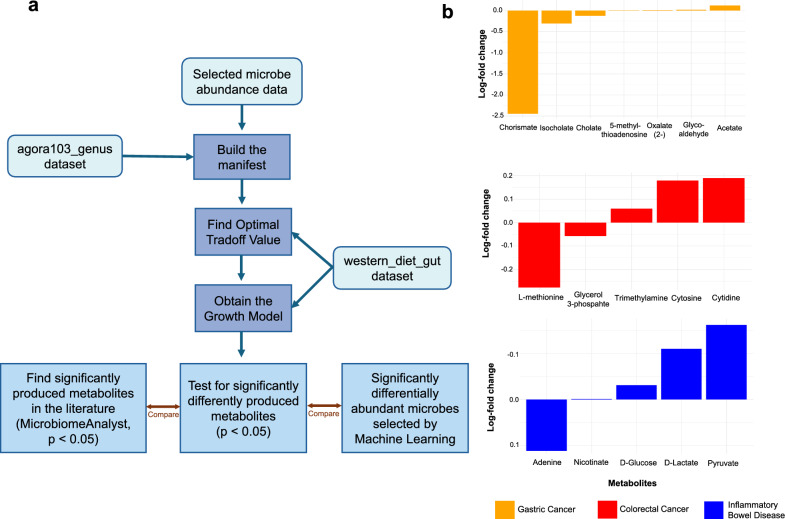
Microbial community model (MICOM) results overview. **a** A summary of the process used to obtain the results. **b** The significantly differentially produced metabolites (p < 0.05) for each disease and their log-fold change abundance, where a positive change represents an increase in cases vs controls (Diseased vs Healthy)

An overview of the process used to obtain these results can be found in Fig. 2a. Of the 59, 82, and 83 initial genera chosen by the machine learning process, which were input into the MICOM manifest, 14, 14, and 5 were recognized by the AGORA database for GC, CRC, and IBD datasets, respectively. The optimal trade-off values for the created models were 0.8, 0.7, and 0.9 (Supplementary Table 18). Initial analysis of differentially produced metabolites between control and case groups identified 6, 7, and 16 metabolites for GC, CRC, and IBD, respectively, all of which are reported in detail within Supplementary Table 19. Of those we deemed significant, 65% of metabolites were of increased abundance in cases vs controls.

Chorismate (p = 0.002), isocholate (p = 0.0002), and cholate (p = 0.02) were decreased in abundance, while 5-methylthioadenosine (p = 0.02), oxalate (p = 0.01), glycolaldehyde (p = 0.01) and acetate (p = 0.01) were increased in abundance, for GC cases versus controls. L-methionine (p = 0.004) and glycerol 3-phosphate (p = 0.008) were in reduced abundance, along with trimethylamine (p = 0.02), cytosine (p = 0.006) and cytidine (p = 0.03) in increased abundance, for CRC cases vs controls (Fig. 2b).

Adenine (p = 0.03) was diminished in IBD cases, whilst nicotinate (p = 0.04), D-glucose (p = 0.04), D-lactate (p = 0.05), and pyruvate (p = 0.04) were elevated, in IBD cases versus controls (Fig. 2b). Consequently, the overlap between the machine learning analysis and MICOM selected metabolites defines cytidine, glycine, and methionine as important for CRC definition, while no overlap occurred for either IBD or GC.

Additionally, however, MICOM identified metabolite derivatives of those identified by the feature selection process, namely glutamate, glycerate N-acetyl-histidine and nicotinic acid for IBD; glycerophosphate for CRC; and 5-methyl-2-deoxycytidine, acetyl CoA, and glycocholate for GC.

MICOM further selected metabolites as differential in one disease, which were significantly identified by the machine learning algorithm for others. For instance, cholate and cytosine were differential for IBD analysis but were selected as significant for GC and CRC, respectively, by MICOM. Similarly, alanine, glutamate, histidine, lactate, and tryptophan were differential for IBD and 1-methyladenosine differential for CRC as chosen by MICOM, while they were selected as important for CRC by the machine learning process. Finally, alanine and nicotinate were selected for IBD, glycerophosphate for CRC, and methionine for both IBD and CRC by MICOM, whereas they were discriminatory for GC in the machine learning models.

MicrobiomeAnalyst also identified similar metabolites as significantly associated with each disease: D-glucose, glycine, histamine, and L-alanine for IBD; L-phenylalanine, L-leucine, azelaic acid, cholic acid, and lactic acid for GC; and isoleucine, methionine, butyric acid, D-glucose, Serine and 3-methylhistidine for CRC.

### Weighted gene co-expression network analysis

For WGCNA, although different co-expressed metabolite modules were identified for GC, CRC, and IBD when analyzed with 45, 72, and 73 metabolites, respectively, no modules were detected for the microbiome-metabolite dataset. This could be explained by the fact that, in contrast to the more stable and evolutionarily conserved gene networks, interactions between the microbiota and metabolites are highly variable and transient. Nevertheless, to determine an appropriate stable configuration for the study, an adequate level of network connectivity was chosen. Furthermore, co-expression networks were shown, emphasizing the modular structures and metabolite interactions in each dataset.

**Fig. 3:**
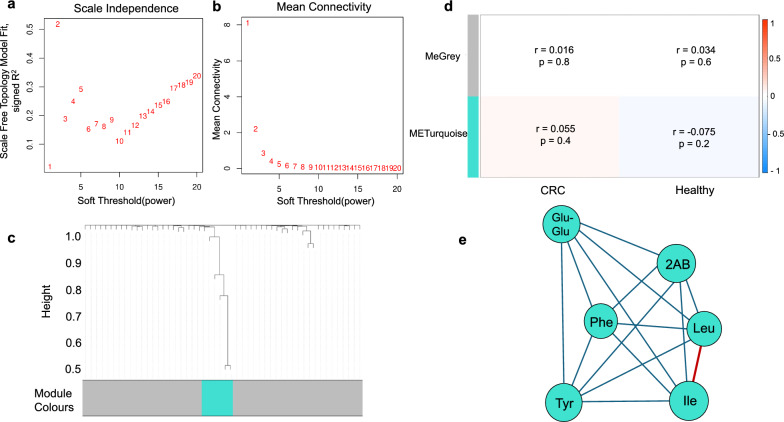
Weighted Gene Co-expression Network Analysis (WGCNA) for CRC. **a** This plot shows the scale-free topology model fit (R^2^) versus soft-thresholding power (β). The highest R^2^ is 0.1847 at β = 9, indicating a weak but improving fit to the scale-free topology as β increases. **b** This plot displays how the mean connectivity decreases with increasing β. At β = 9, the mean connectivity is low, reflecting network sparsification while retaining some structural connections. **c** Shows a hierarchical clustering dendrogram of metabolites, where branches represent clusters of similar elements based on their co-expression. The height (Y-axis) indicates the dissimilarity between clusters, with smaller heights representing higher similarity. The horizontal bar below the dendrogram represents module assignments. The turquoise color indicates elements grouped into a co-expression module, while grey represents elements that were not assigned to any module due to low correlation or lack of clustering. **d** This heatmap represents the correlation between module eigengenes and traits (Case and Control, where Case = CRC and Control = Healthy). Each cell contains the correlation coefficient and its p-value with color intensity indicating the correlation’s strength and direction (red for positive, blue for negative). The grey module, containing unassigned elements, shows a very weak positive correlation with CRC (r = 0.016, p = 0.8) and Healthy (r = 0.034, p = 0.6), both of which are statistically insignificant. The turquoise module, containing co-expressed elements, shows a weak positive correlation with CRC (r = 0.055, p = 0.4) and a weak negative correlation with Healthy (r = − 0.075, p = 0.2), neither of which are significant. This suggests no strong relationship between module expression and CRC. **e** The turquoise nodes in the network visualisation represent metabolites within the turquoise module, characterized by strong co-expression connections. The edges connecting turquoise nodes reflect the strength of co-expression: red edges represent higher strongly co-expression interactions, and blue edges indicate lower co-expression interactions. Metabolites like"Leu,"and"Ile"are central to this cluster, potentially functioning as hub metabolites coordinating module activity

For the CRC metabolite dataset, a power of β = 9 was selected, yielding an R^2^ of 0.1847 (Fig. 3a&b). Among the co-expressed metabolite modules identified, the turquoise module stood out as a unique cluster strongly associated with CRC which included metabolites like Glu-Glu, isoleucine (Ile), 2 AB, phenylalanine (Phe), leucine (Leu), and tyrosine (Tyr) (Fig. 3c).

A heatmap visualising the correlation between module eigengenes (ME) and clinical characteristics (CRC vs. Healthy, denoted as cases vs. controls revealed that the turquoise module showed a modest positive association with CRC cases (correlation = 0.055, p = 0.4) and a slight negative correlation with healthy controls. (correlation = − 0.075, p = 0.2) (Fig. 3d).

The network diagram illustrated the dense interconnections among metabolites in the turquoise module, with leucine (Leu) and isoleucine (Ile) occupying central positions as hub metabolites (Fig. 3e). Metabolites from other modules, which were not part of this co-expression cluster, were represented by grey nodes.

For the GC metabolite dataset, the turquoise module was significantly associated with GC in the WGCNA analysis, developed with a soft threshold power of β = 9 (scale-free topology R^2^ = 0.1947) (Supplementary Fig. 7a, b). The turquoise module, represented a distinct cluster of co-expressed metabolites, including N-acetylglucosamine-1-phosphate, agmatine, 5-aminolevulinate, N8-acetylspermidine, inosine, nicotinamide, S-adenosylmethionine (SAM), dihydrouracil, and uracil are the nine metabolites that made up this module (Supplementary Fig. 7c). A heatmap revealed correlation between clinical characteristics (GC vs. Healthy) and MEs. The turquoise module showed a modest positive correlation with the Healthy group (correlation = 0.073, p = 0.5) and a slight negative correlation with the GC group (correlation = − 0.13, p = 0.2), suggesting its potential role in distinguishing between cases and controls (Supplementary Fig. 7 d). In the network diagram, turquoise-colored nodes represent metabolites from the turquoise module, which is strongly linked to GC, while grey nodes denote metabolites from other modules. Key metabolites such as SAM, inosine (Ino), uracil (Ura), and N8-acetylspermidine (Agm) were moderately interconnected, highlighting their central roles in the turquoise module's metabolic network.

Finally, for IBD, a soft threshold power (β = 10) was selected to construct a scale-free topology network, balancing sparsity and robustness, with a model fit (R^2^) of 0.235 (Supplementary Fig. 8a, b). Hierarchical clustering based on topological overlap identified distinct co-expression modules, with the turquoise module emerging as the most biologically significant (Supplementary Fig. 8c). The turquoise module showed a positive correlation with IBD, despite a modest eigengene-disease status association (correlation = 0.056, p = 0.4) (Supplementary Fig. 8 d).

This module comprised ten metabolites moderately associated with IBD, including sebacate, deoxycholic acid, 7-ketodeoxycholate, thiamine, cholestenone, pyridoxamine, undecanedionate, lithocholic acid, 4-hydroxy-3-methylacetophenone, and urobilin.

## Discussion

Early GID diagnosis is essential for both preventing disease progression and developing efficient treatment plans that can enhance patient survival. Traditionally, each GID relies on its own'gold standard’ diagnostic methods, such as endoscopy, medical imaging, and biopsies. While effective, these methods are often invasive, costly, might carry the risk of radiation exposure [62–64], and may not always detect the disease at an early stage. To tackle these challenges, researchers have explored biomarkers for early and accurate detection of GC, CRC, and IBD individually using genomic, transcriptomic, microbiome, and metabolomic datasets. Given that GIDs are often interconnected, the presence of one condition can increase the risk of developing another. This study examines whether microbes and metabolites linked to one disease could serve as early indicators for diagnosing others.

### Biomarkers in gastric cancer

Biomarker identification largely depends on stable feature selection to ensure reliability. To achieve this, we employed multiple feature selection methods, including RFECV and LASSO-based selection, prioritizing top-ranked features with the highest discriminative scores and ensuring that selected features were not highly correlated with one another, with the help of the Spearman correlation map. Combining the selected microbes and metabolites for GC provided optimal performance scores of AUC > 0.8 (0.63–1.00) across all three machine learning models. Since the goal of this project is to use the primary GC model to predict CC and IBD and vice versa, we applied the same selected biomarkers to IBD and CC datasets. The results revealed that GC biomarkers might also be relevant for IBD, with all models achieving AUC > 0.7 (0.66–1.00). However, while the IBD models demonstrated high accuracy, precision, specificity, and F1 scores, their sensitivity was comparatively lower, indicating a reduced ability to identify all true positive cases. This trade-off essentially reflects the model’s tendency to prioritize minimizing false positives over maximizing true positives. Similar observations were made by Hodgkiss et al. [65], who also noted low sensitivity in IBD prediction models.

In contrast, although the GC biomarkers performed well for IBD, they performed poorly in predicting CRC, with most models showing an AUC just above 0.58 (0.51–0.58), except for the Random Forest model, which achieved an AUC of 0.63 (0.57–0.69).

To reinforce our findings in the literature, we observed that the microbes associated with GC belonged to three major phyla, which included *Firmicutes*, *Bacteroidota*(also known as *Bacteroidetes*), and *Actinobacteria*. Tseng et al. [66] reported that these bacterial phyla were abundant in patients who had recently undergone gastrectomy, which aligns with our observations, as the data were collected from GC patients post-gastrectomy.

In our study, we identified microbes that belong to the *Lachnospiraceae* family, which is known to participate in the production of acetic acid and butyric acid. A reduction in the abundance of *Lachnospiraceae* was associated with altered lipid metabolism, increased inflammation, and malignancy in GC [67–69]. Additionally, the *Muribaculaceae* family showed a positive correlation to amino acid and glucose metabolism pathways related to GC [70, 71].

Studies reported conflicting effects of *Ruminococcus* on GC, with some studies indicating that certain species can be beneficial in reducing the risk of CRC and stabilising the intestinal barrier [72], while certain species of *Ruminococcus* can potentially increase the risk of developing GC [73]. Additionally, *Centipeda*, a microbe found significant in GC in our study, has been strongly associated with cancer virulent *H.pylori* [74]. Similarly, *Cutibacterium*, another significant microbe in GC, has been studied extensively for its role in promoting tumor formation in renal cell carcinomas [75] and has also been found to be abundant in GC as well [76, 77].

Regarding metabolites, dihydrouracil ranked highly in LASSO feature importance. Although not directly involved in GC causation, dihydrouracil plays a key role in pyrimidine metabolism, which, when disrupted, can lead to cancerous lesions [78–80]. Shentu et al. [81] revealed that taurine exhibits dual roles in GC disease progression, promoting tumor growth in immunodeficient mice while inhibiting it in immunocompetent mice.

Moreover, Sinha et al. [82], in their meta-analysis regarding the effects of taurine on CRC, noted that most studies report an increase in the taurine levels associated with the disease. $\gamma$-Butyrobetaine, which serves as a precursor in the formation of Trimethylamine N-Oxide (TMAO) by gut flora like *Firmicutes* and Actinobacteria, which is known to be associated with the development of GC [83, 84]. Secondary bile acid glycocholate, also known as glycocholic acid, was seen to be increased in patients with GC [85] and UC [86]. In contrast, MICOM analysis identified decreased derivatives of the bile acid glycocholate, namely chorismate, isocholate, and cholate, in GC cases vs controls. This was based on the metabolite flux predicted from the differential microbes. This could highlight that the increased abundance of bile acids is not due to their differential production by our selected microbes but may be due to other alterations in downstream cellular mechanisms [87]. Methionine sulfoxide, another key metabolite from GC machine learning analysis, was produced at lower levels by the selected microbes for IBD and CC cases in MICOM analysis. The promising use of this metabolite as an adjuvant researched in all three disorders [88–90] gives insight into the potential of this pathway as a link in their pathology.

Furthermore, acetate, a derivative involved in the acetyl CoA pathway that has been linked to cancer cell growth [91], was predicted to be significantly increased in GC samples based on microbial abundance in MICOM. Additional metabolites associated with the differential taxa from machine learning analysis and an increase in GC disease by MICOM included oxalate and glycolaldehyde linked with the development of renal dysfunction in GC patients [92], and GC metastasis [93], respectively.

### Biomarkers in colorectal cancer

The combined model incorporating both microbes and metabolites for the CRC dataset achieved an AUC > 0.7 (0.57–0.86) across XGBoost, Random Forest, and LASSO models. When this model was applied to the GC and IBD datasets, its performance was notably better for GC, achieving an AUC > 0.7 (0.58–0.93) across all three models. This suggests that the biomarkers identified for CRC may also be relevant for GC.

Microbes identified from the CRC dataset were predominantly from the phylum *Firmicutes*, followed by *Bacteroidetes*, *Fusobacteriota*, *Actinobacteriota*, and *Deinococcota*. Interestingly, although 7 of the 13 microbes belonged to the *Firmicutes* phylum, studies have reported conflicting findings regarding its abundance in CRC patients. Some studies suggest a reduction in *Firmicutes* abundance in CRC patients, particularly those species involved in butyrate production [94, 95]. In contrast, other studies indicate an increase in *Firmicutes* along with *Bacteroidetes*, *Fusobacteriota*, *Actinobacteriota*, and *Deinococcota*, which have been found to be more prevalent and abundant in CRC patients [96–99].

At the genus level, *Fusobacterium* emerged as a key bacterium frequently associated with periodontal diseases [100]. Recognized for its pro-inflammatory properties, *Fusobacterium* was found to be more abundant in advanced stages of both CRC and GC [101, 102]. Additionally, *Fusobacterium* is linked to the production of hydrogen sulfide, which plays a role in the synthesis of sulfur-containing amino acids, a process implicated in the initiation of CRC [103]. This highlights its potential role in driving disease progression.

Furthermore, species from the genus *Enterococcus*, known for their production of reactive oxygen species (ROS), were observed in both colonic and gastric epithelial tissues, implicating their role in epithelial damage and tumorigenesis in both CRC and GC [104, 105]. Genera like *Selenomonas* [106, 107] and *Thermus* [97, 108] were also seen to be abundant in patients with both CRC and GC, reinforcing their potential as biomarkers. 

In our study, we identified isoleucine, a branched-chain amino acid (BCAA), which exhibits a complex and dual role in tumor progression. Some studies on CRC suggest that isoleucine promotes tumor growth by participating in biosynthetic pathways as an intermediate in the TCA cycle that supplies energy and contributes to oncogenic mutations [109]. Additionally, Ren et al. [110] demonstrated that *Clostridium symbiosum* produces BCAAs such as isoleucine, which enhance cholesterol synthesis, a process implicated in CRC progression. In contrast, other studies on CRC [111] and GC [112] reported a protective role for isoleucine, suggesting it may inhibit tumor formation and emphasizing its dual role in cancer biology, warranting further investigation. 

In the WGCNA analysis conducted, no strong interconnections between metabolites were observed in CRC. However, network analysis identified leucine and isoleucine as hub metabolites, suggesting their involvement in a tightly interconnected metabolic network. A recent study in mouse models found that the breakdown of leucine and isoleucine played a crucial role in the development of CRC, with elevated levels of these metabolites found in CRC tumor tissues compared to normal tissues. This indicates that impaired breakdown of BCAAs supports cancer cell proliferation by providing essential nutrients for tumor growth. In CRC, the normal degradation of the BCAAs is disrupted due to the downregulation of proteins involved in their breakdown. As a result, these amino acids accumulate, promoting cancer cell metabolism and growth [113]. A study by Wang et al. [114], revealed that B cells enriched in CRC tissues with transforming growth factor-β1(TGF-β1) dominant regulatory phenotypes are driven by leucine nutritional preferences and accelerate CRC growth. Leucine promotes tumor evasion by inducing leucine-tRNA-synthetase-2 expressing B cell (LARS B) which inhibits mitochondrial NAD + regeneration and oxidative metabolism, leading to increased TGF-β1 production. 

The metabolite nicotinamide also showed interesting results. While essential for normal cellular metabolism, its abundance in CRC may confer a survival advantage to cancer cells, as suggested by Jabbari et al. [115]. Similarly, targeting nicotinamide metabolism in GC patients may improve prognosis [116]. Other metabolites demonstrated disease relevance as well. Decanoate (capric acid), a medium-chain fatty acid, showed anticarcinogenic properties in CRC [117]. Elevated levels of N1, N8-diacetylspermidine were observed in CRC patients [118], while metabolites such as guanine [119, 120] and γ-guanidinobutyrate [121, 122] were identified in both CRC and GC as potential biomarkers. Cytidine, which is strongly linked to gut inflammation in CRC [123], was proposed to be increased in CRC cases according to both MICOM and machine learning analysis, whereas glycine, which has the potential to decrease CRC tumor volume and vascularization [124], was decreased in abundance. Additionally, metabolites associated with the selected microbes in CRC analysis, such as trimethylamine and cytosine, were predicted to be of higher abundance in CRC samples. An increase of trimethylamine was also observed in CRC patients by Guo et al. [125] and linked to dysbiosis by Chan et al. [126], while the increased activity of cytosine activated pathways has been associated with CRC progression [127]. Furthermore, metabolites identified as differential for CRC in early machine learning analysis such as glutamate and alanine were selected as differential in IBD patients in MICOM analysis. Glutamate is strongly related to the maintenance of the mucosal lining, with its disruption contributing to IBD and gastrointestinal cancer [128], while amino acids such as alanine and leucine are key to mucosal healing after destruction. Their low abundance can lead to issues in both CRC and IBD [129, 130].

### Biomarkers in inflammatory bowel disease

The combined model scores for IBD, which incorporated both microbes and metabolites, showed satisfactory results with AUC values > 0.84 (0.71–0.98). However, when applied to both GC and CRC, the predictions were suboptimal. While GC showed slightly better performance, with AUC scores > 0.60 (0.53–0.76) for Random Forest and LASSO, other performance metrics did not perform as well.

At the phylum level, most of the microbes identified in IBD belonged to the *Firmicutes* and *Actinobacteriota*. These phyla, typically involved in the breakdown of short-chain fatty acids (SCFAs) into butyrate and other beneficial products, were reported to be reduced in the gut microbiomes of IBD patients in a study by Tsai et al. [131] Conversely, Santoru et al. [132] observed an increase in these phyla in IBD patients, highlighting the need for further investigation by looking more into the genera and the family levels. As many of the microbes were unclassified at the genus level, we traced them to the family level classifications. Genera such as *Bariatricus*, *Butyribacter*, *Limivivens*, and *UBA11774*, all members of the *Lachnospiraceae* family, were consistently found to be decreased in abundance in IBD patients compared to healthy controls in multiple studies [133, 134]. Interestingly, certain species within *Lachnospiraceae* have been linked to tumor progression in GC [67]. At the genus level, we examined *Faecalibaculum*, which was identified in IBD was recognized for its probiotic properties, SCFA production, and production of indole-3-lactic acid, which helps reduce colonic inflammation and repair the gut epithelial barrier [135, 136] However, Chen et al. [137] confirmed the presence of *Faecalibaculum* in cases of small bowel disease and severe gastritis. In terms of metabolites in IBD, urobilin, and acetyl-arginine have been identified as possible biomarkers in previous studies [138, 139]. Elevated levels of glyceric acid (glycerate) have been observed in the fecal samples of IBD patients, likely due to the breakdown of triacylglycerols released from the colon mucosa, which contributes to metabolic disruptions such as acidosis [132]. Notably, glycerate has also been implicated in GC, where it participates in the glycolysis pathway, providing energy to support tumor growth. This aligns with the Warburg effect, a metabolic hallmark of cancer, where cancer cells preferentially rely on glycolysis for energy production, even under oxygen-rich conditions, to fuel their rapid proliferation and survival [140, 141]. Furthermore, D-glucose and glutamate were significantly increased in IBD samples of MICOM analysis, increasing the promotion of Th17 cell differentiation and the activation of transforming growth factor β [142], which further promotes cell proliferation and inflammation and weakens the auto-immune response [143]. Heightened levels of lactate in IBD patients were also found by Song et al. [144], in addition to our MICOM analysis, while contrastingly, many other studies have determined lactate is beneficial for the intestinal barrier and a potential therapeutic target for IBD [145, 146]. This difference may be due to the fact that the differential microbes used to produce the MICOM model are highly lactate producing in normal circumstances, but may not have been in the guts of our patient cohort. Furthermore, nicotinate and pyruvate, also overabundant in IBD samples of the MICOM analysis, have been under investigation as therapeutic targets, with inhibitors of their metabolic pathways showing promising results [147, 148].

Metabolites with protective roles in the context of GIDs were also identified in this study. Inosine and carnosol, for instance, have been identified for their anti-inflammatory properties and for maintaining the intestinal barrier in both IBD and GC [149–152]. Similar patterns were observed in MICOM analysis. Moreover, adenine, which was found to be underabundant in IBD, activates receptors responsible for anti-inflammatory macrophages [153], potentially by inhibiting the tumour necrosis factor-α (TNF-α) induced interleukin-8 secretion pathway [154].

### Machine learning for microbiome and metabolomic disease classification

In our study, Random Forest consistently emerged as the top-performing model for both individual microbiome, and combined models for GC, CRC, and IBD indicating high discriminatory power between the healthy and diseased samples. While high performance scores alone do not always correlate with better results, Random Forest is widely used in microbiome studies due to its ability to model complex nonlinear relationships between features and outcomes, as well as its robustness to noise [155]. For example, Gao et al. [156] demonstrated that the Random Forest based pipeline achieved the best classification performance for CRC prediction compared to other models, with performance metrics improving as the number of decision trees increased in the meta-dataset. Similarly, Zheng et al. [157], identified Random Forest as a reliable diagnostic model for distinguishing between healthy controls, CD, and UC patients based on gut microbiome data with AUC = 0.81 (0.80–0.82). Appiah et al. [158] also showed that Random Forest could accurately identify potential microbial signatures that distinguish healthy controls from GC samples.

On the other hand, LASSO emerged as the top model for metabolomic data in GC and CRC due to its ability to handle high dimensional data and shrink less important variable coefficients to zero, effectively selecting the most relevant metabolites for disease prediction.

Supporting this, Chen et al. [159] developed a 10-metabolite GC diagnostic model using LASSO, achieving a sensitivity of 0.9. Similarly, Sun et al. [160] successfully used LASSO regression to distinguish between healthy individuals and CRC patients with AUC = 0.96 based on plasma and fecal metabolites. Overlapping metabolites identified by the machine learning analysis and MICOM predicted models suggest potential links between differential microbes and the metabolites in disease. Furthermore, these interaction models provide the potential to design optimized fecal microbial transplant (FMT) treatments that not only address dysbiosis but also incorporate metabolite supplementation or degradation to enhance disease therapy [161].

## Shared pathological mechanisms and biomarker insights in GC, CRC, and IBD

GC, CRC, and IBD exhibit distinct yet overlapping clinical and pathological characteristics that are often driven by shared clinical risk factors and underlying disease mechanisms. For instance, GC often presents with non-specific symptoms that include acid reflux, dysphagia, abdominal pain, bloating, indigestion, weight loss, and melaena [162]. The most common subtype, gastric adenocarcinoma, is known to frequently metastasize to the lymph nodes and liver. Several risk factors increase the likelihood of developing GC, including *H. pylori* infection, gastroesophageal reflux disease (GORD), family history, and poor diet [163, 164]. Furthermore, *H. pylori* is also linked to mucosa-associated lymphatic tissue (MALT) lymphoma in the stomach, primarily due to chronic inflammation and bacterial virulence factors [165].

CRC, predominantly in the form of colonic adenocarcinoma, presents with hallmark cancer symptoms such as weight loss, fatigue, and anemia [166]. However, it also presents more specific signs like rectal bleeding (haematochezia), tenesmus, and changes in bowel habits.

Certain inherited genetic conditions, such as Lynch syndrome (Hereditary Nonpolyposis Colon Cancer) and familial adenomatous polyposis (FAP), significantly increase the risk of developing CRC [16]. Unlike some cancers, CRC tends to metastasize more widely, often affecting the lungs, liver, and peritoneum [167]. Chronic diarrhea mixed with blood in stools is a hallmark of IBD and the following inflammation, especially in UC, predisposes patients to CRC through inflammation-induced DNA damage and immune dysregulation [7, 8, 32]. IBD and GC share immune dysregulation features, including imbalances in T-helper cells(*Th1, Th17*), which may also contribute to CRC progression [168–170]. CRC and GC both harbor mutations in key oncogenes like PIK3 CA and exhibit dysregulation in signaling pathways like Wnt/β-catenin contributing to cancer progression and resistance to therapy [171–174]^.^

Moreover, chronic inflammation in IBD creates a pro-tumorigenic environment by increasing oxidative stress, which leads to DNA damage, p53 mutations, and microsatellite instability, persistent inflammatory cytokine signaling, ultimately facilitating the progression from dysplasia to CRC [175]. Understanding these shared mechanisms could aid in developing therapeutic approaches that target dysbiosis, promote beneficial microbes and metabolites, and modulate the harmful ones to help predict GIDs more effectively.

## Limitations

One of the key limitations of our study was the failure to account for confounding factors such as age, gender, BMI, and diet in our predictive models. These variables can significantly influence model performance and introduce bias if not properly controlled [176]. Secondly, there was variability in the methods used for data collection, preprocessing, and analysis across different datasets and countries. This inconsistency can significantly impact the performance of the models [177], especially when integrating multiple datasets from diverse disease types. For instance, during validation, we observed suboptimal performance in some of our models, which we attribute to these methodological differences. To address this, establishing standardized protocols for data collection and analysis could greatly enhance the robustness and accuracy of machine learning models in future studies. Another limitation arose from the way we categorized diseases.

In this study, CD and UC were grouped under IBD, and different stages of CRC were analyzed collectively without distinguishing the adenoma specific data. This may have resulted in the loss of insights specific to individual disease subtypes or stages, potentially affecting the performance of our models. Additionally, while we used an independent dataset to validate the predictive power of our machine learning models, the validation process was limited to a small subset of the identified microbes and metabolites. This constraint highlights the need for larger, more diverse validation datasets to ensure the generalisability of our findings. Finally, our use of the MICOM model, which simulates microbial community interactions, was limited by the scope of the AGORA database. Since the database only includes a fraction of known microbes, only 6–24% of the differentially abundant microbes in our study could be incorporated into the disease models. This limitation underscores the importance of further research into the functional roles of unclassified microbes and their metabolic pathways. By expanding our understanding of these microbes, we can fill critical gaps in model creation and improve their predictive accuracy.

## Future work

The use of a longitudinal study could enhance this study, as it would allow us to follow microbial and metabolomic changes in the gut at different stages, and we could gain a much clearer picture of how the disease starts and develops. Additionally, adding more GI disorder types can be incorporated in the future to develop a complete diagnostic model for different disorders with high specificity and sensitivity. This deeper understanding could help improve early detection and lead to more effective treatment strategies down the line.

## Conclusion

Findings from our study suggest that differential microbes and metabolites associated with GC could also serve as potential biomarkers for predicting IBD. Interestingly, when we examined the microbes and metabolites linked to CRC, we found that they had a stronger predictive performance for GC than for IBD. These observations point to the possibility of overlapping disease pathways and biological mechanisms, supporting the idea that microbes and metabolites from one GID can be used to predict another. To further validate these findings, we cross referenced our identified microbes and metabolites with existing literature which reinforced the notion that certain biomarkers are shared across different GIDs. This opens up the possibility of developing diagnostic tools that could enhance our understanding and treatment of GIDs.

## Supplementary Information

Additional file 1.

Additional file 2.

## Data Availability

This paper analyzes existing, publicly available data. Information on access and associated publications is provided in Table 1. The Python and R packages used in this study can be found in Supplementary Table 1, and the underlying code generated for this study has been deposited in Figshare and can be accessed via this link: https://doi.org/10.6084/m9.figshare.28464557.

## abbreviations

GIDs: Gastrointestinal disorders
GC: Gastric cancer
CRC: Colon cancer
IBD: Inflammatory bowel disease
XGBoost: EXtreme gradient boosting
LASSO: Least absolute shrinkage and selection operator
MICOM: Microbial community
WGCNA: Weighted Gene Co-expression Network Analysis
ROC-AUC: Receiver Operator Curve-Area Under the Curve
PCA: Principal components analysis
PcoA: Principal coordinates analysis
CD: Crohn’s disease
UD: Ulcerative colitis
