# Supplementary material for: Deciphering microbial and metabolic influences in gastrointestinal diseases-unveiling their roles in gastric cancer, colorectal cancer, and inflammatory bowel disease
Source: J Transl Med. 2025 May 16;23:549. doi: 10.1186/s12967-025-06552-w (PMC12085054; doi:10.1186/s12967-025-06552-w)
Supplement: Supplementary file 1 — Additional file 1. [file 12967_2025_6552_MOESM1_ESM.pdf]

Deciphering Microbial and Metabolic Influences in Gastrointestinal Diseases-Unveiling Their Roles in Gastric Cancer, Colorectal Cancer, and Inflammatory Bowel Disease

Daryll Philip[1], Rebecca Hodgkiss[2], Swarnima Kollampallath Radhakrishnan[2], Akshat Sinha[2], Animesh Acharjee[1,2,3,4*]

[1]Cancer and Genomic Sciences, University of Birmingham, Dubai, UAE

[2]Cancer and Genomic Sciences, University of Birmingham, Birmingham, UK

[3]Centre for Health Data Research, University of Birmingham, Birmingham, UK

[4]Institute of Translational Medicine, University Hospitals Birmingham NHS, Foundation Trust, B15 2TT, UK

*Correspondence

Dr. Animesh Acharjee

University of Birmingham, B15 2TT, UK

E-mail: a.acharjee@bham.ac.uk

Phone: +44 121 414 7012

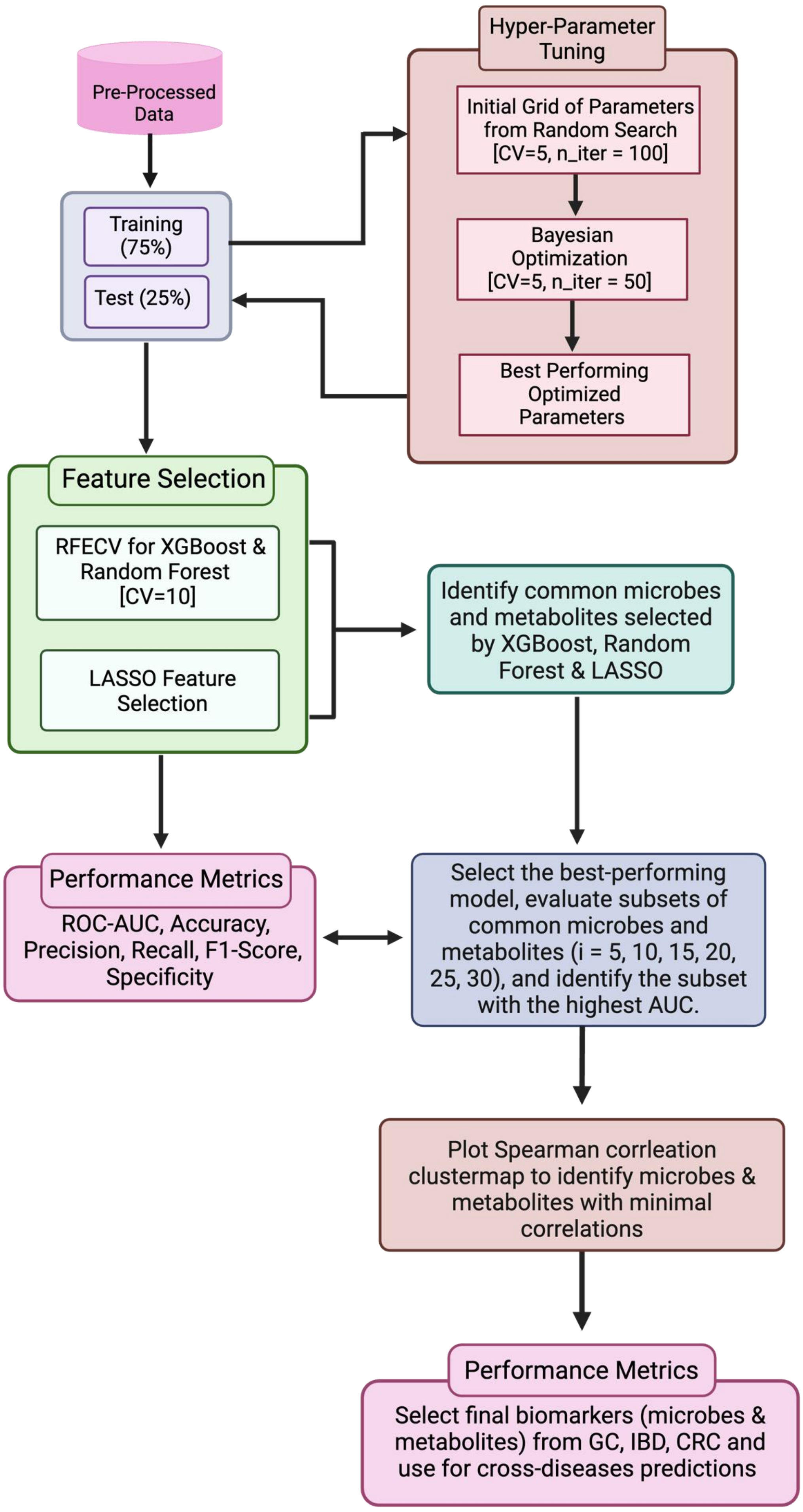

Supplementary Figure 1. Machine learning workflow. The machine learning pipeline for XGBoost, Random Forest, and LASSO incorporates hyperparameter tuning, feature selection, and model evaluation using performance metrics for gastric cancer(GC), colorectal cancer(CRC), and inflammatory bowel disease(IBD). Machine learning models identify shared microbes and metabolites. The best performing model is optimized with the top 5–30 features selected based on ROC-AUC scores. A Spearman correlation cluster map reveals feature relationships and removes highly correlated features, and the final biomarker performance is evaluated. XGBoost: Extreme Gradient Boosting, ROC-AUC: receiver operator curve – area under the curve.

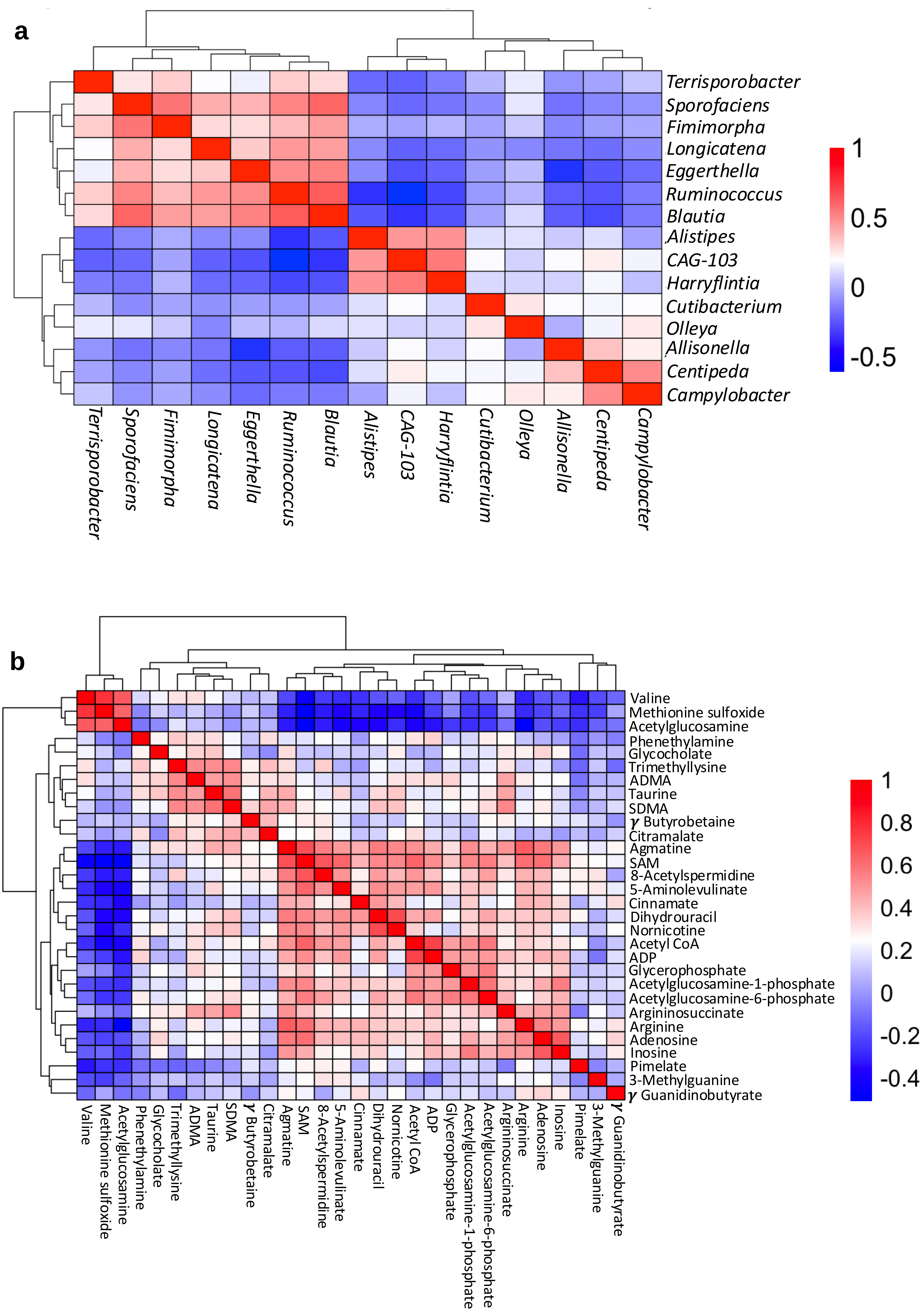

Supplementary Figure 2. Spearman correlation cluster map with hierarchical clustering for GC. (a) Top 15 microbes chosen by the Random Forest model based on Gini importance scores and **(b)** top 30 metabolites chosen by the LASSO model. From the cluster maps 6 microbes and 8 metabolites that did not have a high correlation to other features were chosen as biomarkers for further analysis.

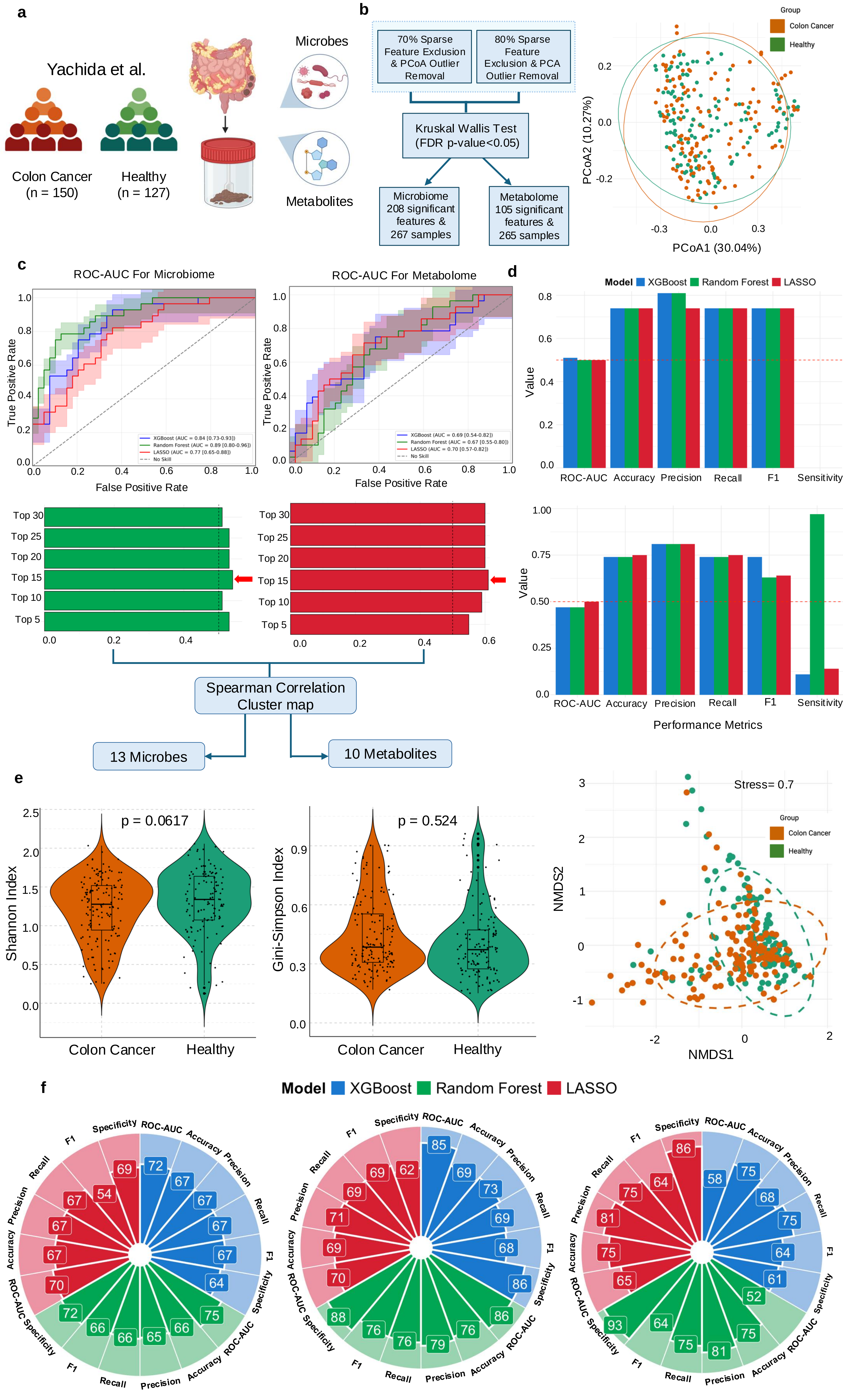
a
Yachida et al.
Colon Cancer (n = 150)
Healthy (n = 127)
Microbes
Metabolites
b
70% Sparse Feature Exclusion & PCoA Outlier Removal
80% Sparse Feature Exclusion & PCA Outlier Removal
Kruskal Wallis Test (FDR p-value<0.05)
Microbiome 208 significant features & 267 samples
Metabolome 105 significant features & 265 samples
Group
Colon Cancer
Healthy
PCoA2 (10.27%)
PCoA1 (30.04%)
c
ROC-AUC For Microbiome
True Positive Rate
False Positive Rate
XGBoost (AUC = 0.84 [0.73-0.93])
Random Forest (AUC = 0.89 [0.80-0.96])
LASSO (AUC = 0.77 [0.65-0.88])
No Skill
ROC-AUC For Metabolome
XGBoost (AUC = 0.69 [0.54-0.82])
Random Forest (AUC = 0.67 [0.55-0.80])
LASSO (AUC = 0.70 [0.57-0.82])
No Skill
Top 30
Top 25
Top 20
Top 15
Top 10
Top 5
Spearman Correlation Cluster map
13 Microbes
10 Metabolites
d
Model XGBoost Random Forest LASSO
Value
ROC-AUC
Accuracy
Precision
Recall
F1
Sensitivity
Performance Metrics
e
p = 0.0617
Shannon Index
p = 0.524
Gini-Simpson Index
Colon Cancer
Healthy
Stress= 0.7
NMDS2
NMDS1
f
Model XGBoost Random Forest LASSO
ROC-AUC
Accuracy
Precision
Recall
F1
Specificity

Supplementary Figure 3. Microbiome-metabolome machine learning for cross-disease predictions in CRC. (a) Fecal microbiome and metabolome data from CRC patients (orange) and healthy individuals (green) were obtained from Yachida et al. **(b)** Data preprocessing workflow highlighting key microbes, metabolites, and samples selected for machine learning, alongside a principal coordinates analysis (PCoA) plot used for outlier removal. **(c)** The receiver operator curve – area under the curve (ROC-AUC) for microbiome and metabolome data across models: XGBoost (blue), Random Forest (green), and LASSO (red). Bar graph showing the best-performing model (microbiome-Random Forest, metabolome-LASSO) based on the highest AUC-ROC score, highlighting the optimal number of features. The selection includes 13 microbial and 10 metabolite features identified through Spearman cluster map analysis. **(d)** Validation performance metrics depicted by bar plots for microbiome and metabolome analysis evaluated using the dataset from Kim et al. **(e)** Alpha diversity analysis, visualised using violin plots comparing healthy and diseased patients, assessed using the Shannon Index and Gini-Simpson index. FDR-corrected p-values ($p<0.05$) revealed no statistically significant differences within the two groups for both indices. Beta diversity was evaluated using non-metric multidimensional scaling (NMDS) based on Jaccard distances, with the stress value confirming no significance between healthy and diseased patients. **(f)** Circular bar plots illustrate the performance scores of the three models trained using combined microbiome and metabolome data from CRC patients. Key biomarkers from the CRC dataset were identified in GC and IBD datasets. CRC-trained models were applied to predict GC and IBD outcomes respectively.

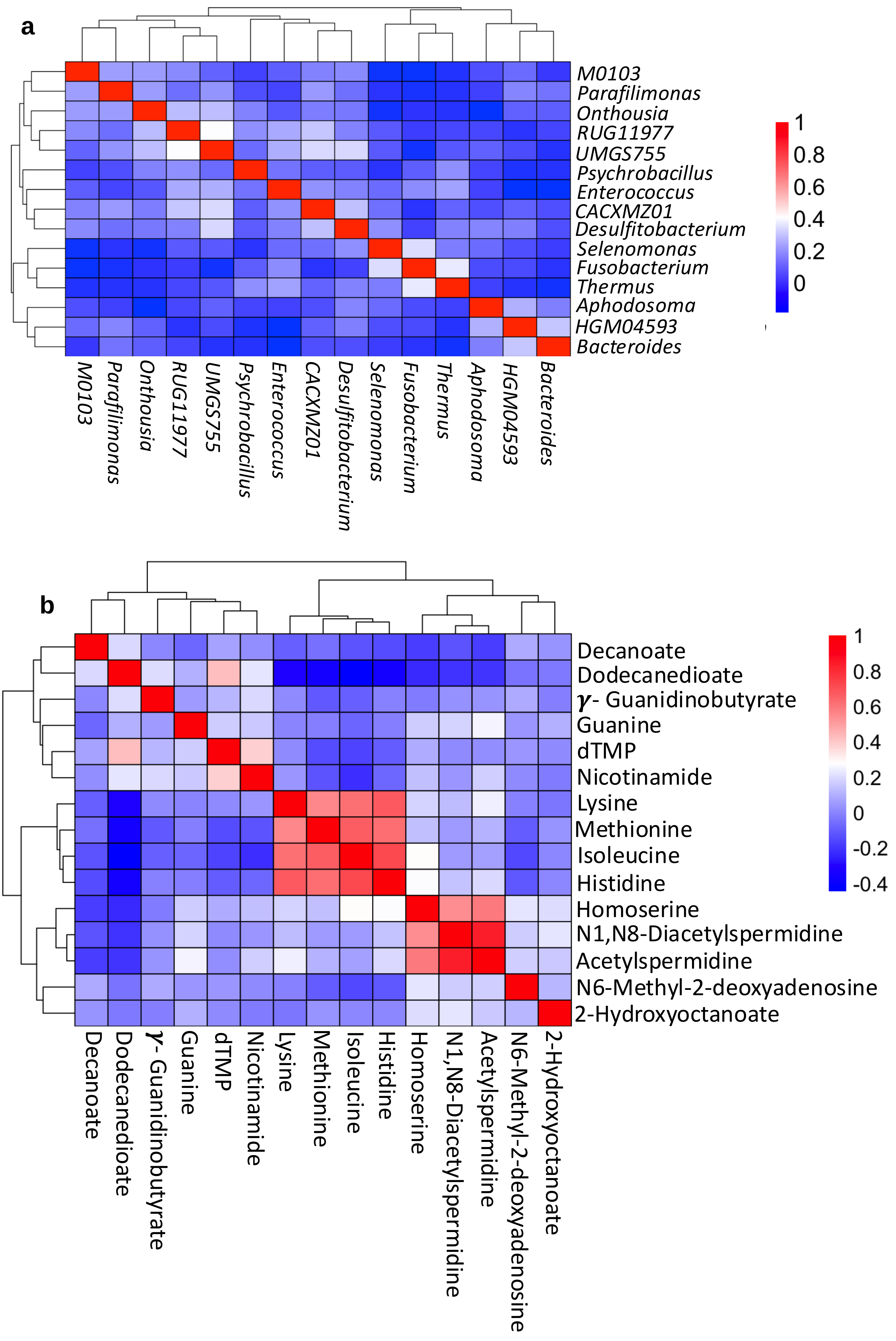

Supplementary Figure 4. Spearman correlation cluster map with hierarchical clustering for CRC (a) Top 15 microbes chosen by the Random Forest model based on Gini-importance scores and **(b)** top 15 metabolites chosen by the LASSO model. From the cluster maps 13 microbes and 10 metabolites that did not have a high correlation to other features were chosen as biomarkers for further analysis.

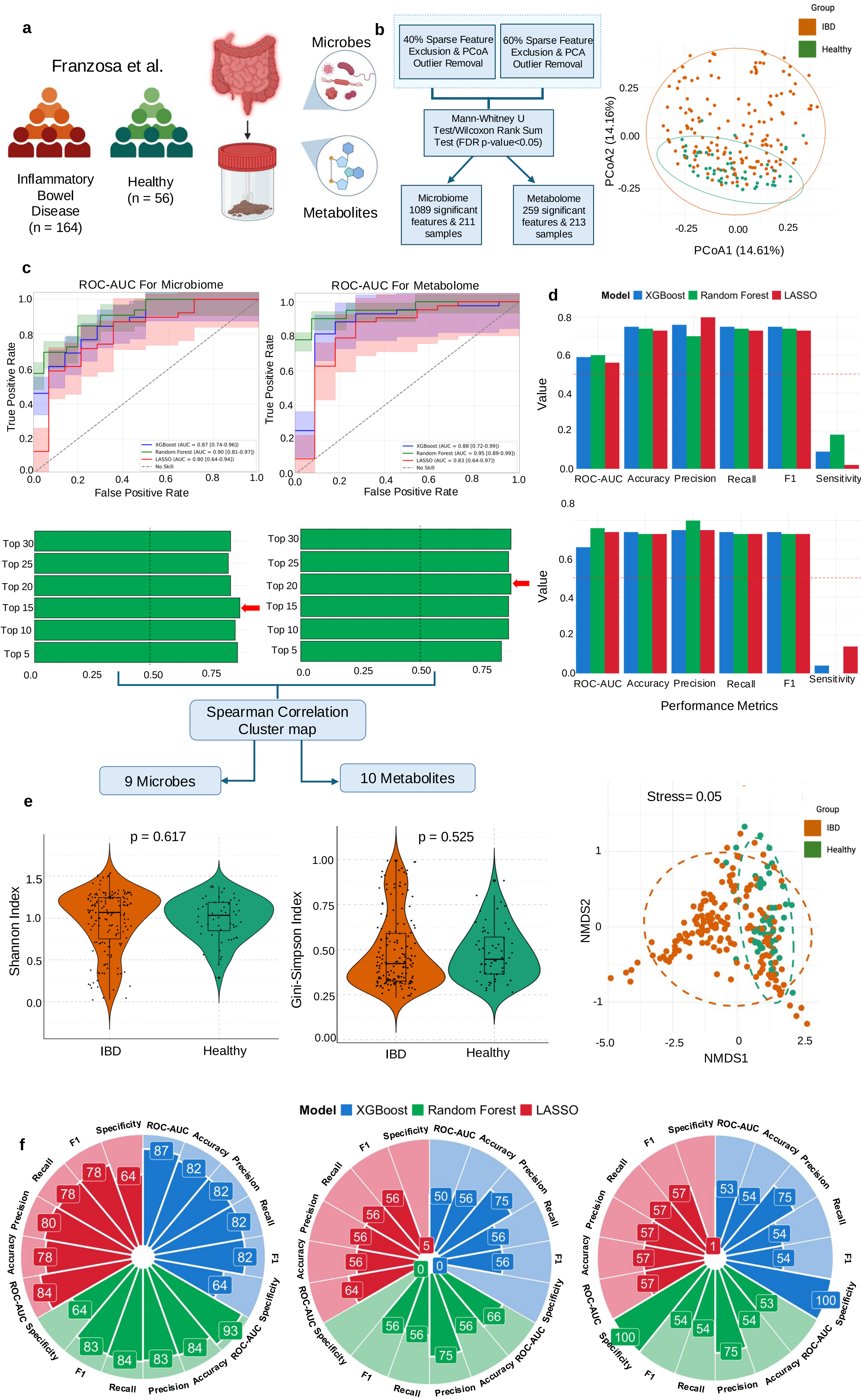
a
Franzosa et al.
Inflammatory Bowel Disease (n = 164)
Healthy (n = 56)
Microbes
Metabolites
b
40% Sparse Feature Exclusion & PCoA Outlier Removal
60% Sparse Feature Exclusion & PCA Outlier Removal
Mann-Whitney U Test/Wilcoxon Rank Sum Test (FDR p-value<0.05)
Microbiome 1089 significant features & 211 samples
Metabolome 259 significant features & 213 samples
Group
IBD
Healthy
PCoA2 (14.16%)
PCoA1 (14.61%)
c
ROC-AUC For Microbiome
ROC-AUC For Metabolome
True Positive Rate
False Positive Rate
XGBoost (AUC = 0.87 [0.74-0.96])
Random Forest (AUC = 0.90 [0.81-0.97])
LASSO (AUC = 0.80 [0.64-0.94])
No Skill
XGBoost (AUC = 0.88 [0.72-0.99])
Random Forest (AUC = 0.95 [0.89-0.99])
LASSO (AUC = 0.83 [0.64-0.97])
No Skill
Top 30
Top 25
Top 20
Top 15
Top 10
Top 5
Spearman Correlation Cluster map
9 Microbes
10 Metabolites
d
Model XGBoost Random Forest LASSO
Value
ROC-AUC Accuracy Precision Recall F1 Sensitivity
Performance Metrics
e
p = 0.617
Shannon Index
p = 0.525
Gini-Simpson Index
IBD
Healthy
Stress= 0.05
NMDS2
NMDS1
f
Model XGBoost Random Forest LASSO
ROC-AUC
Accuracy
Precision
Recall
F1
Specificity

Supplementary Figure 5. Microbiome-metabolome machine learning for cross-disease predictions in IBD. (a) Fecal microbiome and metabolome data from IBD patients (orange) and healthy individuals (green) were obtained from Franzosa et al. **(b)** Data preprocessing workflow highlighting the key microbes, metabolites, and samples selected for machine learning analysis. **(c)** The receiver operator curve – area under the curve (ROC-AUC) for microbiome and metabolome data across models: XGBoost (blue), Random Forest (green), and LASSO (red). Bar graph showing the best-performing model (microbiome-Random Forest, metabolome-Random Forest) based on the highest AUC-ROC score, highlighting the optimal number of features. The selection includes 9 microbial and 10 metabolite features identified through Spearman cluster map analysis. **(d)** Validation performance metrics of the optimal features depicted by bar plots for microbiome and metabolome analysis were evaluated using the dataset from the Integrative Human Microbiome Project (iHMP). **(e)** Alpha diversity for microbes was visualised with violin plots comparing healthy and IBD patients using the Shannon and Gini-Simpson indices. FDR-corrected p-values ($p < 0.05$) showed no significant differences within both groups. Beta diversity was evaluated using non-metric multidimensional scaling (NMDS) based on Jaccard distances, with the stress value confirming statistical significance between healthy and diseased patients. **(f)** Circular bar plots illustrate the performance scores of the three models trained using combined microbiome and metabolome data from IBD patients. Key biomarkers from the IBD dataset were identified in the GC and CRC datasets. IBD-trained models were applied to predict GC and CRC outcomes respectively.

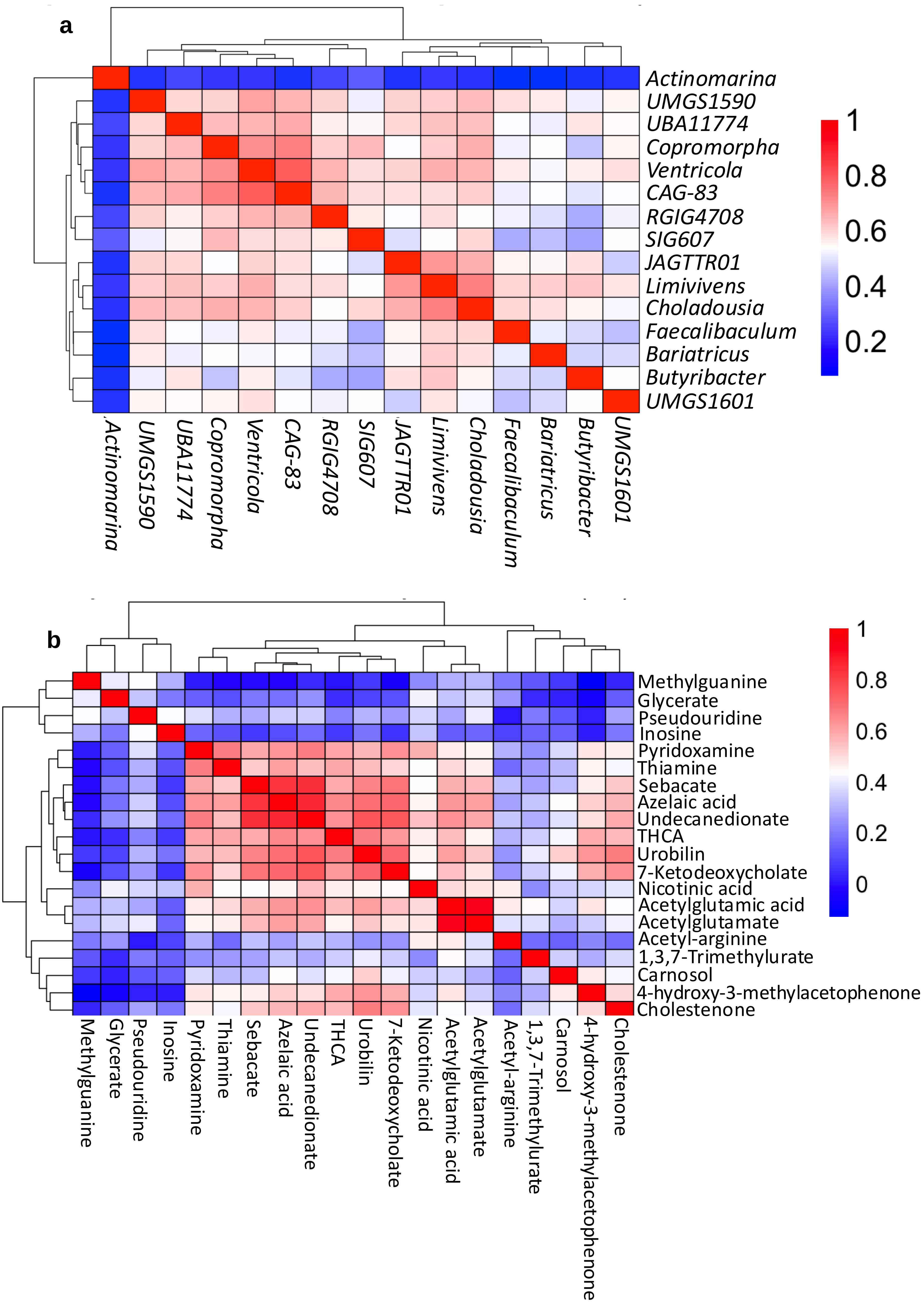

Supplementary Figure 6. Spearman correlation cluster map with hierarchical for IBD. (a) Top 15 microbes and **(b)** top 20 metabolites chosen by the Random Forest models based on Gini importance scores. From the cluster maps 9 microbes and 10 metabolites that did not have a high correlation to other features were chosen as biomarkers for further analysis.

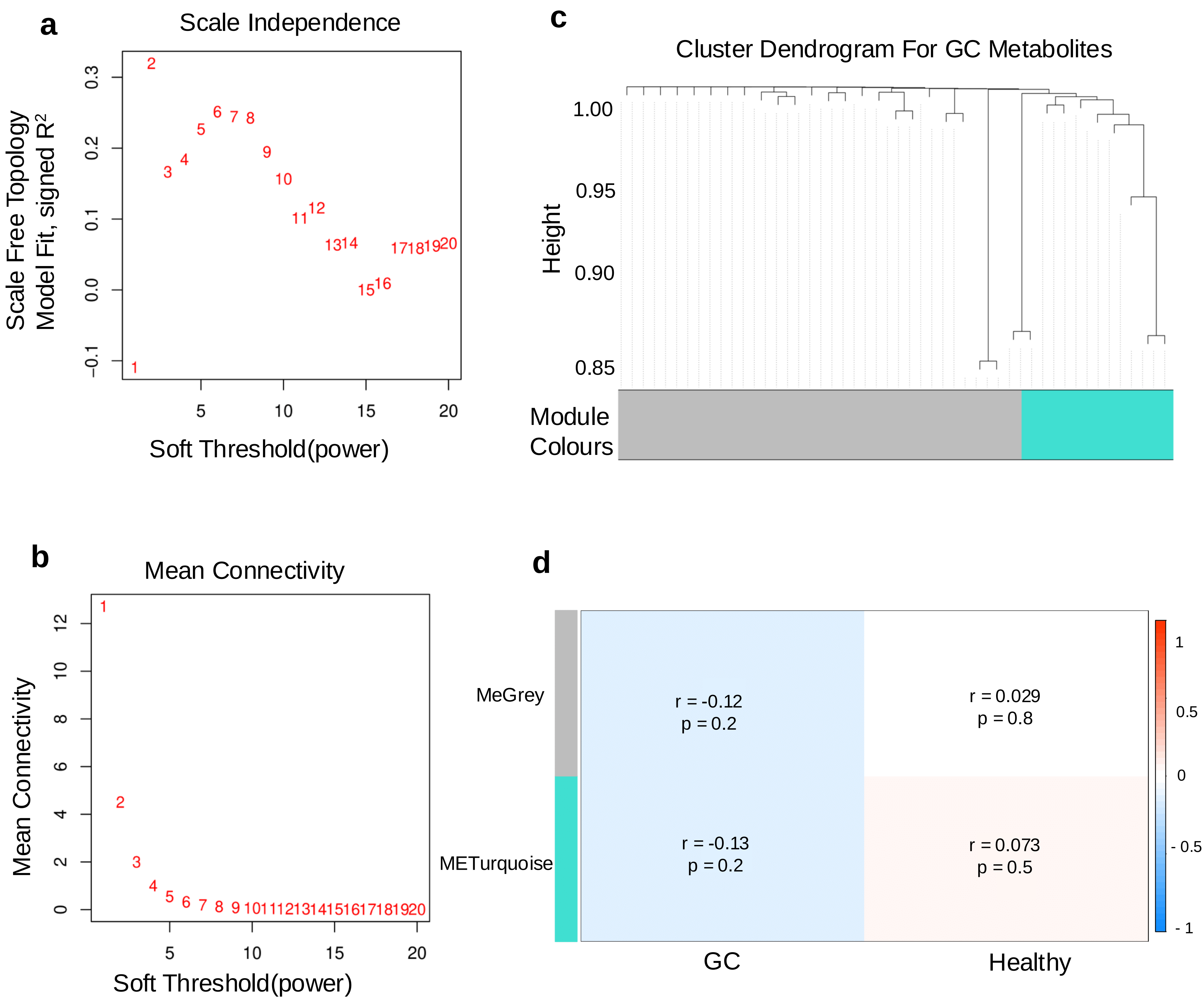

Supplementary Figure 7. Weighted Gene Co-expression Network Analysis (WGCNA) for GC. (a) This plot shows the scale-free topology model fit (R^2) versus soft-thresholding power (β). The highest R^2 is 0.1947 at $\beta = 9$, indicating the network does not strongly conform to a scale-free topology but improves slightly with increasing β. **(b)** This plot displays how the mean connectivity decreases with increasing β. At $\beta = 9$, the connectivity is low, suggesting that the network is sparse but still retains some structure. **(c)** Shows a hierarchical clustering dendrogram of metabolites, where branches represent clusters of similar elements based on their co-expression. The height (Y-axis) indicates the dissimilarity between clusters, with smaller heights representing higher similarity. The horizontal bar below the dendrogram represents module assignments. The turquoise colour indicates elements grouped into a co-expression module, while grey represents elements that were not assigned to any module due to low correlation or lack of clustering. **(d)** This heatmap displays the correlation between module eigengenes and traits or conditions (Case and Control, where Case = GC and Control = Healthy). Each cell shows the correlation coefficient and its p-value, with the colour intensity indicating the correlation's strength and direction (blue for negative, red for positive). The grey module, which contains unassigned elements, shows a weak negative correlation with Case ($r = -0.12$, $p = 0.2$) and a very weak positive correlation with Control ($r = 0.029$, $p = 0.8$). The turquoise module, containing co-expressed elements, shows a weak negative correlation with Case ($r = -0.13$, $p = 0.2$) and a weak positive correlation with Control ($r = 0.073$, $p = 0.5$). Both modules exhibit weak and statistically insignificant correlations with the traits, indicating minimal association between module expression and the Case/Control conditions.

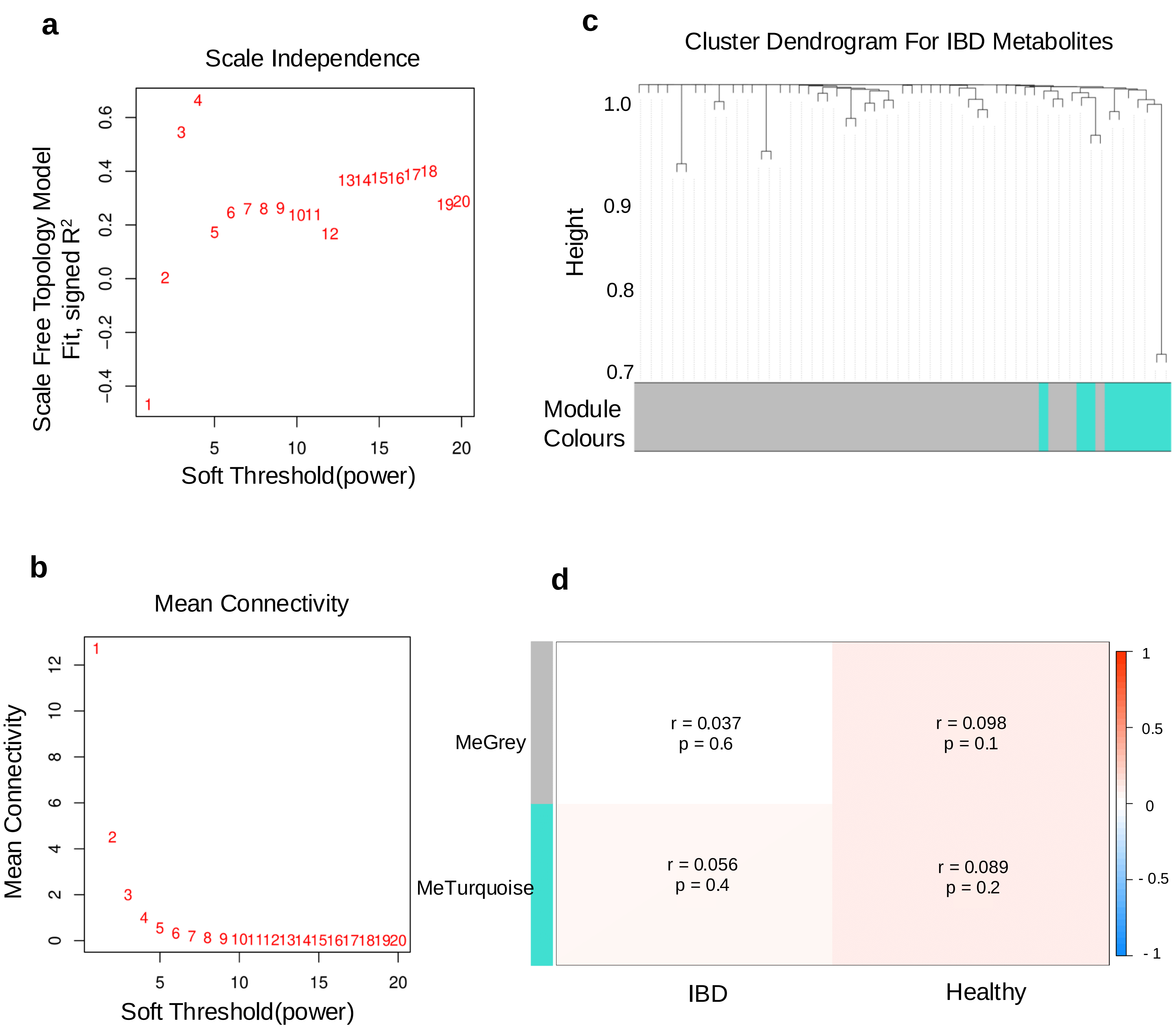

Supplementary Figure 8. Weighted Gene Co-expression Network Analysis (WGCNA) for IBD. (a) This plot shows the scale-free topology model fit (R^2) versus soft-thresholding power (β). Although the R^2 values are low (e.g., ~0.2 at $\beta = 10$), increasing β improves the scale-free topology fit gradually. **(b)** This plot displays how the mean connectivity decreases with increasing β, reflecting network sparsification. A balance is needed to ensure the network retains meaningful structure while approximating a scale-free topology. **(c)** Shows a hierarchical clustering dendrogram of metabolites, where branches represent clusters of similar elements based on their co-expression for IBD. The height (Y-axis) indicates the dissimilarity between clusters, with smaller heights representing higher similarity. The horizontal bar below the dendrogram represents module assignments. The turquoise colour indicates elements grouped into a co-expression module, while grey represents elements that were not assigned to any module due to low correlation or lack of clustering. **(d)** This heatmap shows the correlation between module eigengenes and traits (Case and Control, where Case=IBD and Control=Healthy). Each cell displays the correlation coefficient and its p-value, with the colour intensity reflecting the strength and direction of the correlation (red for positive, blue for negative). The grey module, which contains unassigned elements, shows weak correlations with both Case ($r = 0.037$, $p = 0.6$) and Control ($r = 0.098$, $p = 0.1$). The turquoise module, containing co-expressed elements, also shows weak correlations with both Case ($r = 0.056$, $p = 0.4$) and Control ($r = 0.089$, $p = 0.2$). Both modules exhibit weak correlations with traits, suggesting minimal association between module expression and the IBD/Healthy conditions.
