# Supplementary material for: Deciphering microbial and metabolic influences in gastrointestinal diseases-unveiling their roles in gastric cancer, colorectal cancer, and inflammatory bowel disease
Source: J Transl Med. 2025 May 16;23:549. doi: 10.1186/s12967-025-06552-w (PMC12085054; doi:10.1186/s12967-025-06552-w)
Supplement: Supplementary file 2 — Additional file 2. [file 12967_2025_6552_MOESM2_ESM.pdf]

Deciphering Microbial and Metabolic Influences in Gastrointestinal Diseases-Unveiling Their Roles in Gastric Cancer, Colorectal Cancer, and Inflammatory Bowel Disease

Daryll Philip[1], Rebecca Hodgkiss[2], Swarnima Kollampallath Radhakrishnan[2], Akshat Sinha[2], Animesh Acharjee[1,2,3,4*]

[1]Cancer and Genomic Sciences, University of Birmingham, Dubai, UAE

[2]Cancer and Genomic Sciences, University of Birmingham, Birmingham, UK

[3]Centre for Health Data Research, University of Birmingham, Birmingham, UK

[4]Institute of Translational Medicine, University Hospitals Birmingham NHS, Foundation Trust, B15 2TT, UK

*Correspondence

Dr. Animesh Acharjee

University of Birmingham, B15 2TT, UK

E-mail: a.acharjee@bham.ac.uk

Phone: +44 121 414 7012

Supplementary Table 1. Computational and statistical packages. Summary of the R and Python packages utilised in this study.

Softwares and Packages		
R (v4.3.2)	R Core Team	https://www.r-project.org/
FactoMineR	Lê et al.	https://github.com/husson/FactoMineR
Factoextra	Kassambara et al.	https://github.com/kassambara/factoextra
vegan	Oksanen et al.	https://github.com/vegandevs/vegan
BioCManager	Morgan et al.	https://github.com/Bioconductor/BiocManager
ape	Paradis et al.	https://github.com/cran/ape
phyloseq	McMurdie and Holmes	https://github.com/joey711/phyloseq
naniar	Tierney et al.	https://github.com/njtierney/naniar
glue	Hester et al.	https://github.com/tidyverse/glue
reshape2	Hadley Wickham	https://github.com/hadley/reshape
devtools	Wickham et al.	https://github.com/r-lib/devtools
tidyverse	Wickham et al.	https://github.com/tidyverse
pheatmap	Raivo Kolde	https://github.com/raivokolde/pheatmap
WGCNA	Langfelder and Horvath	https://github.com/Lindseynicer/WGCNA_tutorial
Python (v3.9.6)	Van Rossum et al.	https://www.python.org/
scikit-learn	Pedregosa et al.	https://github.com/scikit-learn/scikit-learn
pandas	Wes McKinney	https://github.com/pandas-dev/pandas
NumPy	Travis Oliphant	https://github.com/numpy/numpy
seaborn	Michael Waskom	https://github.com/mwaskom/seaborn
optuna	Akiba et al.	https://github.com/optuna/optuna
xgboost	Tianqi Chen	https://github.com/dmlc/xgboost
matplotlib.pyplot	Hunter et al.	https://github.com/matplotlib/matplotlib
Imbalanced-learn	Lemaître et al.	https://github.com/scikit-learn-contrib/imbalanced-learn
MICOM	Diener et al.	https://github.com/micom-dev/micom

Supplementary Table 2. Baseline demographic characteristics. Table describing the calculations done on the validation datasets. p-values for categorical variables, such as sex, were calculated using Fisher's exact test, while for continuous variables, such as BMI and age, the Mann-Whitney U test was used.

Population Demographic	Gastric Cancer Microbiome Validation (Jaeyun Sung et al.)				Gastric Cancer Metabolite Validation (UK BioBank)				Inflammatory Bowel Disease Validation (iHMP/HMP2)				Colon Cancer Validation (Kim et al.)			
	Total	GC	Healthy	p value	Total	GC	Healthy	p value	Total	IBD	Healthy	p value	Total	CRC	Healthy	p value
**Participants**	50	10	40		46,814	44,378	2,436		382	278	104		138	36	102	
**Sex**																
Male (%)	78	18	60	0.25	53	49.6	3.45	0.9	52.5	50.5	57.7	0.24	82	20	62	0.83
Female (%)	22	2	20		47	45.2	1.75		47.5	49.5	42.3		56	16	40	
**Age**	63 (25-74)	24 (25-29)	65 (62-68)	0.33	61 (43-69)	61 (43-69)	62 (44-75)	0.22	21 (35-42)	23 (17-32)	17 (16-23)	0.12	64.5	64.5	64.5	0.81

Supplementary Table 3. Performance metrics. Scores were calculated along with their 95% confidence intervals for microbiome and metabolome data in GC (Erawijantari et al.), for XGBoost, Random Forest, and LASSO models.

	Data	Classifier	AUC(%)	Accuracy(%)	Precision (%)	Recall(%)	F1(%)	Specificity(%)
**Gastric Cancer (Erawijantari et al.)**	**Microbiome**	XGBoost	0.94 (0.83-1.00)	0.83 (0.66-0.95)	0.71 (0.46-0.92)	1	0.83 (0.63-0.96)	0.71 (0.45-0.93)
		Random Forest	0.96 (0.86-1.00)	0.88 (0.75-1.00)	0.77 (0.50-1.00)	1	0.87 (0.66-1.00)	0.79 (0.53-1.00)
		LASSO	0.83 (0.64-0.97)	0.75 (0.58-0.91)	0.67 (0.38-0.91)	0.8 (0.50-1.00)	0.73 (0.47-0.90)	0.71 (0.46-0.92)
	**Metabolome**	XGBoost	0.92 (0.76-1.00)	0.88 (0.75-1.00)	0.91 (0.70-1.00)	0.83 (0.60-1.00)	0.87 (0.67-1.00)	0.92 (0.73-1.00)
		Random Forest	0.94 (0.81-1.00)	0.83 (0.67-0.96)	0.83 (0.60-1.00)	0.83 (0.60-1.00)	0.83 (0.63-0.96)	0.83 (0.63-1.00)
		LASSO	0.98 (0.91-1.00)	0.92 (0.79-1.00)	0.92 (0.73-1.00)	0.92 (0.73-1.00)	0.92 (0.78-1.00)	0.92 (0.73-1.00)

Supplementary Table 4. Best performing hyperparameters. These parameters are for the highest-scoring models for the microbiome and the metabolome in GC after hypertuning the models.

Gastric Cancer	Machine Learning Model	Hyperparameters	Value
Microbiome	Random Forest	n_estimators	232
		min_samples_split	8
		min_samples_leaf	4
		max_features	log2
		max_depth	19
		bootstrap	False
Metabolome	LASSO	C	31.8
		max_iter	42551
		solver	saga
		tol	0.0006

Supplementary Table 5. Validation performance metrics. Scores were calculated along with their 95% confidence intervals for microbiome data (Jaeyun Sung et al.) and metabolome data (UKBioBank) in GC, for XGBoost, Random Forest, and LASSO models.

	Data	Classifier	AUC(%)	Accuracy(%)	Precision (%)	Recall(%)	F1(%)	Specificity(%)
**Gastric Cancer Validation**	**Microbiome (Jaeyun Sung et al.)**	XGBoost	0.58 (0.41-0.75)	0.80 (0.68-0.90)	0.84 (0.78-0.91)	0.80 (0.68-0.90)	0.71 (0.55-0.85)	0
		Random Forest	0.88 (0.85-0.99)	0.90 (0.82-0.98)	0.90 (0.83-0.98)	0.90 (0.82-0.98)	0.90 (0.78-0.98)	0
		LASSO	0.50 (0.50- 0.50)	0.80 (0.70-0.90)	0.84 (0.79-0.91)	0.80 (0.68-0.90)	0.71 (0.55-0.85)	1
	**Metabolome (UKBioBank)**	XGBoost	0.50 (0.50-0.50)	0.95 (0.95-0.95)	0.95 (0.95-0.95)	0.95 (0.95-0.95)	0.92 (0.92-0.92)	1
		Random Forest	0.50 (0.50-0.50)	0.95 (0.95-0.95)	0.95 (0.95-0.95)	0.95 (0.95-0.95)	0.92 (0.92-0.92)	1
		LASSO	0.50 (0.50-0.50)	0.95 (0.95-0.95)	0.95 (0.95-0.95)	0.95 (0.95-0.95)	0.92 (0.92-0.92)	1

Supplementary Table 6. Performance metrics. Scores were calculated along with their 95% confidence intervals for the combined microbes and metabolites of the GC model and their predictions on IBD and CRC.

Data	Classifier	AUC(%)	Accuracy(%)	Precision (%)	Recall(%)	F1(%)	Specificity(%)
**Gastric Cancer (Erawijantari et al.)**	XGBoost	0.92 (0.79-1.00)	0.79 (0.62-0.92)	0.81 (0.46-0.93)	0.91 (0.70-1.00)	0.80 (0.59-0.95)	0.69 (0.43-0.93)
	Random Forest	0.94 (0.83-1.00)	0.79 (0.62-0.92)	0.71 (0.46-0.93)	0.91 (0.71-1.00)	0.80 (0.58-0.93)	0.69 (0.43-0.91)
	LASSO	0.86 (0.66-1.00)	0.83 (0.71-0.96)	0.90 (0.67-1.00)	0.82 (0.57-1.00)	0.86 (0.67-0.97)	0.92 (0.75-1.00)
**IBD (Franzosa et al.)**	XGBoost	0.73 (0.66-0.79)	0.76 (0.70-0.82)	0.74 (0.71-0.82)	0.76 (0.70-0.93)	0.69 (0.82-0.89)	0.41 (0.40-0.60)
	Random Forest	0.77 (0.71-0.83)	0.76 (0.70-0.82)	0.78 (0.70-0.82)	0.76 (0.70-0.80)	0.68 (0.61-0.90)	0.09 (0.02-0.16)
	LASSO	0.75 (0.68-0.81)	0.76 (0.71-0.82)	0.74 (0.74-0.86)	0.76 (0.71-0.93)	0.74 (0.70-0.89)	0.34 (0.22-0.47)
**Colon Cancer (Yachida et al.)**	XGBoost	0.59 (0.53-0.65)	0.59 (0.53-0.65)	0.60 (0.53-0.68)	0.59 (0.53-0.65)	0.59 (0.53-0.65)	0.08 (0.05-0.25)
	Random Forest	0.63 (0.57-0.69)	0.61 (0.55-0.67)	0.66 (0.59-0.73)	0.61 (0.55-0.67)	0.61 (0.55-0.67)	0.93 (0.89-0.97)
	LASSO	0.58 (0.51-0.65)	0.56 (0.50-0.62)	0.56 (0.42-0.67)	0.56 (0.45-0.57)	0.53 (0.39-0.59)	0.79 (0.73-0.86)

Supplementary Table 7. Best performing hyperparameters. These parameters are for the highest-scoring models for the combined model in GC after hypertuning the models.

Cross-Disease Prediction Using GC Biomarkers	Machine Learning Model	Hyperparameters	Value
GC	Random Forest	n_estimators	612
		min_samples_split	6
		min_samples_leaf	3
		max_features	log2
		max_depth	84
		bootstrap	False

Supplementary Table 8. Performance metrics. Scores were calculated along with their 95% confidence intervals for microbiome and metabolome data in CRC (Yachida et al.), for XGBoost, Random Forest, and LASSO models.

	Data	Classifier	AUC(%)	Accuracy(%)	Precision (%)	Recall(%)	F1(%)	Specificity(%)
**Colon Cancer (Yachida et al.)**	**Microbiome**	XGBoost	0.84 (0.75-0.93)	0.75 (0.67-0.87)	0.77 (0.58-0.92)	0.78 (0.50-0.84)	0.77 (0.55-0.84)	0.85 (0.73-0.95)
		Random Forest	0.89 (0.80-0.96)	0.84 (0.75-0.91)	0.84 (0.70-1.00)	0.84 (0.54-0.88)	0.83 (0.63-0.89)	0.92 (0.88-1.00)
		LASSO	0.77 (0.65-0.88)	0.69 (0.57-0.79)	0.68 (0.44-0.86)	0.69 (0.40-0.74)	0.68 (0.40-0.74)	0.79 (0.67-0.92)
	**Metabolome**	XGBoost	0.69 (0.54-0.82)	0.67 (0.57-0.78)	0.69 (0.59-0.80)	0.67 (0.57-0.78)	0.67 (0.56-0.78)	0.62 (0.46-0.76)
		Random Forest	0.67 (0.55-0.80)	0.67 (0.55-0.78)	0.68 (0.57-0.80)	0.67 (0.57-0.78)	0.67 (0.55-0.78)	0.76 (0.62-0.91)
		LASSO	0.70 (0.57-0.82)	0.66 (0.54-0.76)	0.65 (0.54-0.77)	0.66 (0.54-0.76)	0.65 (0.54-0.76)	0.74 (0.61-0.88)

Supplementary Table 9. Best performing hyperparameters. These parameters are for the highest-scoring models for the microbiome and the metabolome in CRC after hypertuning the models.

Colon Cancer	Machine Learning Model	Hyperparameters	Value
Microbiome	Random Forest	n_estimators	1679
		min_samples_split	7
		min_samples_leaf	2
		max_features	sqrt
		max_depth	None
		bootstrap	True
		criterion	entropy
Metabolome	LASSO	C	4.89
		max_iter	10000
		solver	saga
		tol	0.1

Supplementary Table 10. Validation performance metrics. Scores were calculated along with their 95% confidence intervals for microbiome and metabolome data in CRC (Kim et al.), and for XGBoost, Random Forest, and LASSO models.

	Data	Classifier	AUC(%)	Accuracy(%)	Precision (%)	Recall(%)	F1(%)	Specificity(%)
**Colon Cancer (Kim et al.)**	**Microbiome**	XGBoost	0.51 (0.47-0.54)	0.74 (0.67-0.81)	0.81 (0.78-0.85)	0.74 (0.67-0.81)	0.74 (0.67-0.81)	0
		Random Forest	0.51 (0.47-0.54)	0.74 (0.67-0.81)	0.81 (0.78-0.85)	0.74 (0.54-0.88)	0.74 (0.63-0.89)	0
		LASSO	0.50 (0.50-0.50)	0.74 (0.75-0.91)	0.74 ((0.67-0.81)	0.74 (0.67-0.81)	0.74 (0.67-0.81)	0
	**Metabolome**	XGBoost	0.47 (0.36-0.59)	0.74 (0.67-0.80)	0.81 (0.78-0.84)	0.74 (0.67-0.80)	0.74 (0.67-0.80)	0.11 (0.2-0.14)
		Random Forest	0.47 (0.55-0.80)	0.74 (0.66-0.80)	0.81 (0.78-0.84)	0.74 (0.66-0.80)	0.63 (0.52-0.72)	0.97 (0.82-1.00)
		LASSO	0.50 (0.39-0.61)	0.75 (0.67-0.82)	0.81 (0.78-0.85)	0.75 (0.67-0.82)	0.64 (0.55-0.74)	0.14 (0.03-0.26)

Supplementary Table 11. Performance metrics. Scores were calculated along with their 95% confidence intervals for the combined microbes and metabolites of the CRC model and their predictions on GC and IBD.

Data	Classifier	AUC(%)	Accuracy(%)	Precision (%)	Recall(%)	F1(%)	Specificity(%)
**Colon Cancer (Yachida et al.)**	XGBoost	0.72 (0.60-0.83)	0.67 (0.57-0.79)	0.67 (0.46-0.76)	0.67 (0.54-0.86)	0.67 (0.52-0.77)	0.64 (0.49-0.78)
	Random Forest	0.75 (0.62-0.86)	0.66 (0.54-0.77)	0.65 (0.54-0.77)	0.66 (0.54-0.77)	0.66 (0.53-0.77)	0.72 (0.58-0.87)
	LASSO	0.70 (0.57-0.82)	0.67 (0.56-0.77)	0.67 (0.56-0.79)	0.67 (0.56-0.77)	0.54 (0.43-0.66)	0.69 (0.55-0.82)
**Gastric Cancer (Erawijantari et al.)**	XGBoost	0.85 (0.76-0.92)	0.69 (0.59-0.78)	0.73 (0.64-0.82)	0.69 (0.59-0.78)	0.68 (0.59-0.78)	0.86 (0.74-0.97)
	Random Forest	0.86 (0.77-0.93)	0.76 (0.67-0.84)	0.79 (0.71-0.86)	0.76 (0.67-0.84)	0.76 (0.67-0.84)	0.88 (0.77-0.97)
	LASSO	0.70 (0.58-0.81)	0.69 (0.59-0.78)	0.71 (0.59-0.78)	0.69 (0.59-0.78)	0.69 (0.59-0.78)	0.62 (0.48-0.76)
**IBD (Franzosa et al.)**	XGBoost	0.58 (0.49-0.66)	0.75 (0.69-0.80)	0.68 (0.69-0.80)	0.75 (0.59-0.78)	0.64 (0.57-0.70)	0.61 (0.61-0.61)
	Random Forest	0.52 (0.50-0.50)	0.75 (0.68-0.80)	0.81 (0.79-0.83)	0.75 (0.70-0.80)	0.64 (0.59-0.74)	0.93 (0.78-1.00)
	LASSO	0.65 (0.57-0.72)	0.75 (0.69-0.80)	0.81 (0.79-0.84)	0.75 (0.68-0.80)	0.64 (0.56-0.70)	0.86 (0.74-0.97)

Supplementary Table 12. Best performing hyperparameters. These parameters are the highest-scoring models for the combined model in CRC after hypertuning the models.

Cross-Disease Prediction Using CRC Biomarkers	Machine Learning Model	Hyperparameters	Value
CRC	Random Forest	n_estimators	381
		min_samples_split	9
		min_samples_leaf	2
		max_features	sqrt
		max_depth	162
		bootstrap	True
		criterion	entropy

Supplementary Table 13. Performance metrics. Scores were calculated along with their 95% confidence intervals for microbiome and metabolome data in IBD (Franzosa et al.), for XGBoost, Random Forest, and LASSO models.

	Data	Classifier	AUC(%)	Accuracy(%)	Precision (%)	Recall(%)	F1(%)	Specificity(%)
IBD (Franzosa et al.)	Microbiome	XGBoost	0.87 (0.75-0.96)	0.81 (0.70-0.91)	0.87 (0.76-0.97)	0.87 (0.76-0.97)	0.87 (0.76-0.97)	0.64 (0.38-0.89)
		Random Forest	0.90 (0.81-0.97)	0.83 (0.72-0.92)	0.83 (0.74-0.93)	0.83 (0.72-0.92)	0.83 (0.75-0.93)	0.80 (0.62-0.98)
		LASSO	0.80 (0.63- 0.94)	0.81 (0.70-0.91)	0.87 (0.76-0.97)	0.87 (0.76-0.97)	0.87 (0.78-0.94)	0.64 (0.38- 0.90)
	Metabolome	XGBoost	0.88 (0.71-0.99)	0.83 (0.74-0.93)	0.95 (0.87-1.00)	0.84 (0.72-0.95)	0.89 (0.81-0.95)	0.82 (0.54-1.00)
		Random Forest	0.95 (0.89-0.99)	0.89 (0.80-0.96)	0.90 (0.81-0.96)	0.89 (0.80-0.96)	0.89 (0.81-0.96)	0.85 (0.65-1.00)
		LASSO	0.83 (0.64-0.97)	0.78 (0.67-0.89)	0.92 (0.83-1.00)	0.79 (0.67-0.90)	0.85 (0.76-0.93)	0.73 (0.43-1.00)

Supplementary Table 14. Best performing hyperparameters. These parameters are for the highest-scoring models for the microbiome and the metabolome in IBD after hypertuning the models.

IBD	Machine Learning Model	Hyperparameters	Value
Microbiome	Random Forest	n_estimators	740
		min_samples_split	3
		min_samples_leaf	4
		max_features	sqrt
		max_depth	None
		bootstrap	False
		criterion	entropy
Metabolome	Random Forest	n_estimators	661
		min_samples_split	8
		min_samples_leaf	1
		max_features	sqrt
		max_depth	100
		bootstrap	False
		criterion	gini

Supplementary Table 15. Validation performance metrics. Scores were calculated along with their 95% confidence intervals for microbiome and metabolome data in IBD (iHMP2), and for XGBoost, Random Forest, and LASSO models.

	Data	Classifier	AUC(%)	Accuracy(%)	Precision (%)	Recall(%)	F1(%)	Specificity(%)
**IBD Validation (iHMP)**	**Microbiome**	XGBoost	0.59 (0.48-0.62)	0.75 (0.70-0.79)	0.76 (0.65-0.80)	0.75 (0.70-0.79)	0.75 (0.70-0.79)	0.09 (0.04-0.15)
		Random Forest	0.60 (0.50-0.64)	0.74 (0.68-0.77)	0.70 (0.49-0.80)	0.74 (0.68-0.77)	0.74 (0.68-0.77)	0.18 (0.00-0.20)
		LASSO	0.56 (0.46-0.59)	0.73 (0.69-0.78)	0.80 (0.79-0.83)	0.73 (0.69-0.78)	0.73 (0.57-0.69)	0.02 (0.00-0.19)
	**Metabolome**	XGBoost	0.66 (0.59-0.72)	0.74 (0.69-0.78)	0.75 (0.61-0.82)	0.74 (0.69-0.78)	0.74 (0.69-0.78)	0.04 (0.01-0.08)
		Random Forest	0.76 (0.70-0.81)	0.73 (0.68-0.77)	0.80 (0.78-0.82)	0.73 (0.68-0.77)	0.73 (0.68-0.77)	0
		LASSO	0.74 (0.67-0.80)	0.73 (0.68-0.77)	0.75 (0.61-0.74)	0.73 (0.68-0.77)	0.73 (0.68-0.77)	0.14 (0.08-0.21)

Supplementary Table 16. Performance metrics. Scores were calculated along with their 95% confidence intervals for the combined microbes and metabolites of the IBD model and their predictions on GC and CRC.

Data	Classifier	AUC(%)	Accuracy(%)	Precision (%)	Recall(%)	F1(%)	Specificity(%)
**IBD (Franzosa et al.)**	XGBoost	0.87 (0.75-0.96)	0.82 (0.71-0.91)	0.82 (0.77-0.97)	0.82 (0.79-0.94)	0.82 (0.75-0.96)	0.64 (0.40-0.89)
	Random Forest	0.93 (0.86-0.98)	0.84 (0.73-0.93)	0.83 (0.73-0.93)	0.84 (0.73-0.93)	0.83 (0.73-0.93)	0.64 (0.36-0.88)
	LASSO	0.84 (0.71-0.95)	0.78 (0.67-0.87)	0.80 (0.69-0.90)	0.78 (0.67-0.87)	0.78 (0.68-0.89)	0.64 (0.38-0.88)
**Gastric Cancer (Erawijantari et al.)**	XGBoost	0.5 (0.50-0.50)	0.56 (0.47-0.66)	0.75 (0.47-0.67)	0.56 (0.49-0.68)	0.56 (0.4-0.68)	0
	Random Forest	0.66 (0.54-0.76)	0.56 (0.46-0.66)	0.75 (0.75-0.77)	0.56 (0.46-0.66)	0.56 (0.46-0.66)	0
	LASSO	0.64 (0.53-0.75)	0.56 (0.46-0.66)	0.56 (0.46-0.66)	0.56 (0.46-0.66)	0.56 (0.46-0.66)	0.05 (0.00-0.13)
**Colon Cancer (Yachida et al.)**	XGBoost	0.53 (0.47-0.59)	0.54 (0.48-0.60)	0.75 (0.48-0.76)	0.54 (0.48-0.60)	0.54 (0.49-0.60)	1
	Random Forest	0.53 (0.42-0.55)	0.54 (0.48-0.60)	0.75 (0.53-0.77)	0.54 (0.48-0.60)	0.54 (0.48-0.60)	1
	LASSO	0.57 (0.51-0.63)	0.57 (0.51-0.62)	0.57 (0.50-0.64)	0.57 (0.51-0.62)	0.57 (0.51-0.63)	0.01 (0.00-0.09)

Supplementary Table 17. Best performing hyperparameters. These parameters are the highest-scoring models for the combined model in IBD after hypertuning the models.

Cross-Disease Prediction Using IBD Biomarkers	Machine Learning Model	Hyperparameters	Value
IBD	Random Forest	n_estimators	421
		min_samples_split	11
		min_samples_leaf	1
		max_features	sqrt
		max_depth	55
		bootstrap	False
		criterion	gini

Supplementary Table 18. The percentage of taxa growing for each trade-off value during trade-off optimisation of the MICOM model. The optimal trade-off value, shown in red, was selected by the largest trade-off value that allowed all of the taxa to grow.

Trade-off values	Percentage of Taxa Growing (%)		
	Gastric Cancer	Colorectal Cancer	IBD
**0**	7.1	14.3	40
**0.1**	100	100	100
**0.2**	100	100	100
**0.3**	100	100	100
**0.4**	100	100	100
**0.5**	100	100	100
**0.6**	100	100	100
**0.7**	100	**100**	100
**0.8**	**100**	92.9	100
**0.9**	92.9	78.6	**100**
**1.0**	85.7	71.4	80

Supplementary Table 19. The metabolites selected by the MICOM algorithm as significantly differentially produced between case and control groups. The trade-off value is as chosen as the optimal value. IBD- Inflammatory Bowel Disease. The production flux statistic is the log change abundance of the metabolite concentration. (upward green arrow: increase in abundance, downward red arrow: decrease in abundance).

Metabolite	p-value	q-value	Production Flux Statistic (Case vs Control)
**Colorectal Cancer**	**Trade-off = 0.7**		
Cytidine	0.027	0.411	0.190 ↑
Cytosine	0.006	0.234	0.180 ↑
Glycerol 3-phosphate	0.008	0.234	0.058 ↓
L-methionine	0.004	0.234	0.277 ↓
Trimethylamine	0.023	0.411	0.060 ↑
Trimethylamine n-oxide	0.016	0.362	0.060 ↑
**Gastric Cancer**	**Trade-off = 0.8**		
Acetate	0.009	0.312	0.121 ↑
Cholate	0.016	0.312	0.128 ↓
Chorismate	0.002	0.147	2.445 ↓
Glycolaldehyde	0.014	0.312	0.018 ↑
Isocholate	0.0002	0.028	0.308 ↓
Oxalate(2-)	0.011	0.312	0.010 ↑
5-methylthioadenosine	0.021	0.357	0.009 ↑
**IBD**	**Trade-off = 0.9**		
Adenine	0.031	0.464	0.112 ↓
Carbon Dioxide	0.075	0.464	0.071 ↑
D-alanine	0.103	0.464	0.074 ↑
D-glucose	0.042	0.464	0.031 ↑
D-lactate	0.047	0.464	0.110 ↑
Glycine	0.104	0.464	0.298 ↑
Hydrogen	0.083	0.464	0.201 ↑
L-asparagine	0.099	0.464	0.377 ↑
L-histidine	0.067	0.464	0.011 ↑
L-methionine	0.054	0.464	1.533 ↑
L-proline	0.082	0.464	0.018 ↑
L-tryptophan	0.057	0.464	0.013 ↑
Maltose	0.064	0.464	0.011 ↑
Nicotinate	0.042	0.464	0.001 ↑
Pyruvate	0.044	0.464	0.162 ↑
Water	0.106	0.464	0.067 ↓
